# Hit Identification of New Potent PqsR Antagonists as Inhibitors of Quorum Sensing in Planktonic and Biofilm Grown *Pseudomonas aeruginosa*

**DOI:** 10.3389/fchem.2020.00204

**Published:** 2020-05-04

**Authors:** Fadi Soukarieh, Ruiling Liu, Manuel Romero, Shaun N. Roberston, William Richardson, Simone Lucanto, Eduard Vico Oton, Naim Ruhul Qudus, Alaa Mashabi, Scott Grossman, Sadiqur Ali, Tomás Sou, Irena Kukavica-Ibrulj, Roger C. Levesque, Christel A. S. Bergström, Nigel Halliday, Shailesh N. Mistry, Jonas Emsley, Stephan Heeb, Paul Williams, Miguel Cámara, Michael J. Stocks

**Affiliations:** ^1^School of Life Sciences, University of Nottingham Biodiscovery Institute, University of Nottingham, Nottingham, United Kingdom; ^2^The National Biofilms Innovation Centre, University of Nottingham, Nottingham, United Kingdom; ^3^School of Pharmacy, University of Nottingham Biodiscovery Institute, University of Nottingham, Nottingham, United Kingdom; ^4^Drug Delivery Group, Department of Pharmacy, Uppsala University, Uppsala, Sweden; ^5^Pharmacometrics Group, Department of Pharmaceutical Biosciences, Uppsala University, Uppsala, Sweden; ^6^Institut de Biologie Intégrative et des Systèmes, Université Laval, Quebec City, QC, Canada; ^7^The Swedish Drug Delivery Center, Department of Pharmacy, Uppsala University, Uppsala, Sweden

**Keywords:** *Pseudomonas aeruginosa*, PqsR, MvfR, Pseudomonas quinolone signal (PQS), alkylquinolone, biofilms, quorum sensing inhibition, quorum quenching

## Abstract

Current treatments for *Pseudomonas aeruginosa* infections are becoming less effective because of the increasing rates of multi-antibiotic resistance. Pharmacological targeting of virulence through inhibition of quorum sensing (QS) dependent virulence gene regulation has considerable therapeutic potential. In *P. aeruginosa*, the *pqs* QS system regulates the production of multiple virulence factors as well as biofilm maturation and is a promising approach for developing antimicrobial adjuvants for combatting drug resistance. In this work, we report the hit optimisation for a series of potent novel inhibitors of PqsR, a key regulator of the *pqs* system, bearing a 2-((5-methyl-5*H*-[1,2,4]triazino[5,6-*b*]indol-3-yl)thio) acetamide scaffold. The initial hit compound **7** (PAO1-L IC_50_ 0.98 ± 0.02 μM, PA14 inactive at 10 μM) was obtained through a virtual screening campaign performed on the PqsR ligand binding domain using the University of Nottingham Managed Chemical Compound Collection. Hit optimisation gave compounds with enhanced potency against strains PAO1-L and PA14, evaluated using *P. aeruginosa pqs*-based QS bioreporter assays. Compound **40** (PAO1-L IC_50_ 0.25 ± 0.12 μM, PA14 IC_50_ 0.34 ± 0.03 μM) is one of the most potent PqsR antagonists reported showing significant inhibition of *P. aeruginosa* pyocyanin production and *pqs* system signaling in both planktonic cultures and biofilms. The co-crystal structure of **40** with the PqsR ligand binding domain revealed the specific binding interactions occurring between inhibitor and this key regulatory protein.

## Introduction

*Pseudomonas aeruginosa* is a Gram-negative pathogen able to infect a range of human body sites causing serious tissue damage, blood stream invasion, and systemic dissemination (Strateva and Yordanov, 2009). This opportunistic pathogen is particularly devastating for immuno-compromised patients and a leading cause of death for those with cystic fibrosis (Winstanley et al., 2016). Current treatments for *P. aeruginosa* infections rely mainly on antibiotics inhibiting essential bacterial targets required for survival. These therapies, whilst effective in some cases, impose selective pressures leading to the rapid emergence of resistance, particularly in biofilms (Blair et al., 2015). There are ~50,000 cases of *P. aeruginosa* infections in the USA every year and around 13% are due to multidrug-resistant strains (Ventola, 2015). *Pseudomonas aeruginosa* has developed resistance to most antibiotic classes including aminoglycosides, cephalosporins, fluoroquinolones and even carbapenems (Potron et al., 2015). Therefore, there is an urgent need to develop alternative strategies for effectively treating infections caused by this organism. The pathogenicity of *P. aeruginosa* stems from a wide range of secreted and cell-associated virulence factors (Gellatly and Hancock, 2013). Anti-virulence strategies, through the attenuation of virulence without interfering with bacterial growth, are viewed as a promising alternative approach to combat drug resistance since they exert less selective pressures on the pathogen (Muhlen and Dersch, 2016; Fleitas Martinez et al., 2019).

QS is a bacterial cell-to-cell communication mechanism that allows bacteria to coordinate gene expression in response to population density reflecting the local concentration of extracellular signaling molecules termed autoinducers (AIs). *P. aeruginosa* employs a quorum sensing (QS) network to regulate the production of a wide range of virulence traits including but not limited to, exoproducts such as pyocyanin, HCN, elastase, lectinA, pyoverdine, drug efflux pumps, and factors required for immune evasion (Williams and Camara, 2009). QS also plays a key role in controlling biofilm development and biofilm mediated resistance to antibiotics (Bjarnsholt et al., 2005; Thomann et al., 2016; Maura and Rahme, 2017; Soukarieh et al., 2018a). *Pseudomonas aeruginosa* has three highly interconnected QS systems: two *N*-acylhomoserine lactone (AHL)-dependent QS systems (the *las* and *rhl* systems) and the *Pseudomonas* Quinolone Signal (*pqs*) system which relies on alkylquinolone (AQ)-derived autoinducers (Lee and Zhang, 2015; Whiteley et al., 2017).

The *P. aeruginosa pqs* system uses the LysR-type transcriptional regulator PqsR (also known as MvfR), to control the expression of the *pqsABCDE* operon that encodes the enzymes required for the biosynthesis of 4-hydroxy-2-heptylquinoline (HHQ) which, upon the action of the mono-oxygenase PqsH, is converted to 2-heptyl-3-hydroxy-4-quinolone (PQS). PQS and HHQ interact with the C-terminal ligand binding domain of PqsR, resulting in a conformational change that leads to the activation of the *pqs* operon likely through the interaction of the helix-turn-helix DNA binding domain of this protein with the *pqsA* promoter. This triggers the production virulence factors and secondary metabolites through a range of PqsR-dependent and PqsR-independent mechanisms, some of which involve PqsE (Diggle et al., 2007; Ben Haj Khalifa et al., 2011; Rampioni et al., 2016).

**Figure 1:**
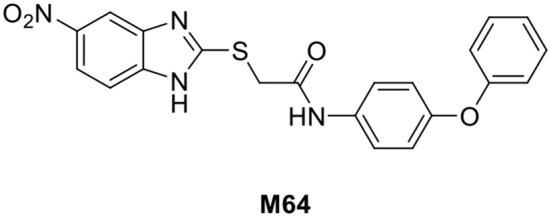
Chemical structure of the M64 PqsR inhibitor.

The *pqs* system is crucial for *P. aeruginosa* pathogenicity and has been regarded as a promising therapeutic target to alleviate antibiotic-resistant infections (Fleitas Martinez et al., 2018). Several attempts to target the *pqs* system with various PqsR inhibitors have previously been reported (Soukarieh et al., 2018a,b). Of these inhibitors, **M64** (Figure 1) was the first PqsR inhibitor to show *in vivo* activity in a mouse lung infection model (Starkey et al., 2014). Due to the lipophilic nature of the PqsR ligand binding site, finding a new series of *pqs* inhibitors, with improved drug-likeness remains a challenge (Ilangovan et al., 2013). In this work, we report the synthesis and biological evaluation of a new series of high potency PqsR inhibitors and demonstrate their ability to inhibit QS in both planktonic and biofilms cultures.

## Materials and Methods

### Data Management and Analysis

Instant JChem was used for Structure Database Management, Search and Prediction, Instant JChem 16.2.15.0 2016, ChemAxon (http://www.chemaxon.com). Sigmoidal dose-response curves and the representation of all data were prepared using GraphPad Prism.

### General Chemistry

Reagents and anhydrous solvents were purchased from Sigma Aldrich, Alfa Aesar and Fisher Scientific, and were used without further purification. Nuclear magnetic resonance: ^1^H-NMR and ^13^C-NMR, were obtained at room temperature using a Bruker AV400 spectrometer operating at 400 MHz. The samples were prepared in deuterated solvent: DMSO-*d*_6_ and CDCl_3_. Chemical shifts (δ) were recorded in ppm and coupling constants (*J*) were recorded in Hz. The spectra were analyzed using MestReNova 12.0.1 software. Mass spectrometry: Analytical HPLC were performed on a Shimadzu UFLCXR system coupled to an Applied Biosystems API2000. Three columns thermostated at 40°C were used. Column one: Phenomenex Gemini-NX 3 μm C18, 50 × 2 mm Column two: Phenomenex Luna 3 μm (PFP2) 110A, 50 × 2 mm. Column three: Waters X terra MS C8 2.5 m, 4.6 × 30 mm. Flow rate 0.5 mL/min. UV detection at 220 (channel 2) and 254 nm (channel 1). Short gradient: Pre-equilibration run for one min at 5% B; then method run: 5–98% solvent B in 2 min, 98% B for 2 min, 98–5% B in 0.5 min then 5% for 1 min. Long gradient: Pre-equilibration run for one min at 5% B; then method run: 5% B for 0.5 min, 10–98% solvent B in 8 min, 98% B for 2 min, 98–5% B in 0.5 min then 5% B for 1 min. Solvent A: 0.1% Formic acid in water; solvent B: 0.1% Formic acid in MeCN. Chromatography: Thin-layer chromatography (TLC) was performed, UV light and standard TLC stains were used to visualize the Merck Silica gel 60 Å F_254_ plates. Compounds were purified via column chromatography using either a Thompson pump or normal phase Interchim Puriflash pre-packed cartridges consisting of 50 μM silica, or a glass column using Merck Geduran silica gel 60 Å (230–240 μm) Column size selected was generally 40–60 times the loading amount.

### General Procedure A: Alkylation of Thiols With *tert-*butyl 2-Bromoacetate (3–4)

*tert*-Butyl 2-bromoacetate (1 mmol) was added dropwise to a suspension of 5-methyl-5*H*-[1,2,4]triazino[5,6*-b*]indole-3-thiols (**1–2**) (Sharma et al., 2014) (1 mmol) and triethylamine (1 mmol) in anhydrous toluene (10 mL) at 0°C under N_2_. The reaction mixture was allowed to slowly warm up to room temperature and stirred for 3 h and was quenched with water (5 mL) and stirred at room temperature for further 5 min. The mixture was then diluted with water (30 mL) and then extracted with EtOAc (20 mL × 3). The organic layers were combined and washed with brine and dried over Na_2_SO_4_. The crude compound was purified by column chromatography.

***tert*****-Butyl 2-((5-methyl-5*H*-[1,2,4]triazino[5,6*-b*]indol-3-yl)thio)acetate (3)**. The title compound was prepared from 5-methyl-5*H*-[1,2,4]triazino[5,6*-b*]indole-3-thiol (**1**) (0.40 g, 1.83 mmol), *tert*-butyl 2-bromoacetate (0.280 g, 1.83 mmol) and triethylamine (0.185 g, 1.83 mmol) according to general procedure A. The crude product was purified by column chromatography (petroleum ether/EtOAc 4:1) to give a white solid (0.37 g, 62%). ^1^H NMR (400 MHz, DMSO-*d*_6_) δ 8.36 (d, *J* = 7.6 Hz, 1H), 7.79 (d, *J* = 4.1 Hz, 2H), 7.51 (dq, *J* = 8.0, 4.3 Hz, 1H), 4.10 (s, 2H), 3.83 (s, 3H), 1.42 (s, 9H). ^13^C NMR (101 MHz, CDCl_3_) δ 168.04, 166.84, 146.28, 141.42, 141.14, 130.74, 122.90, 122.12, 118.01, 109.84, 82.07, 34.54, 28.03, 27.22. LCMS *m/z* calc for C_16_H_18_N_4_O_2_S [M+H]^+^: 330.2, found 330.4 with *t*_R_ 2.91 min, purity 95%.

***tert***-**Butyl 2-((8-bromo-5-methyl-5*H*-[1,2,4]triazino[5,6*-b*]indol-3-yl)thio)acetate (4)**. The title compound was prepared from 8-bromo-5-methyl-5*H*[1,2,4]triazino[5,6*-b*]indole-3-thiol (**2**) (0.15 g, 0.53 mmol), *tert*-butyl 2-bromoacetate (0.081 g, 0.53 mmol) and triethylamine (0.53 g, 0.53 mmol) according to general procedure A. The crude product was purified by column chromatography (petroleum ether/EtOAc 4:1) to give a yellow solid (0.20 g, 88%): ^1^H NMR (400 MHz, DMSO-*d*_6_) δ 8.52 (d, *J* = 2.0 Hz, 1H), 7.94 (dd, *J* = 8.7, 2.0 Hz, 1H), 7.78 (d, *J* = 8.7 Hz, 1H), 4.11 (s, 2H), 3.81 (s, 3H), 1.42 (s, 9H), ^13^C NMR (101 MHz, CDCl_3_) δ 167.92, 167.77, 146.62, 140.30, 140.13, 133.28, 125.09, 119.92, 116.10, 111.39, 82.22, 34.61, 28.02, 27.41. LCMS *m/z* calc for C_16_H_17_BrN_4_O_2_S [M+H]^+^: 408.0, found 408.3 with *t*_R_ 3.05 min, purity 95%.

### General Procedure B: *tert*-butyl Ester Deprotection (5–6)

Intermediates (**3–4**) were dissolved in DCM (3 mL/mmol), 4M HCl in 1,4-dioxane (excess, 2 mL/mmol) was added to the suspension. The mixture was then allowed to stir at room temperature overnight. The solvent was removed under vacuum to yield a light yellow solid. The crude product was washed with diethyl ether and DCM and was used directly for next steps without further purification.

**2-((5-Methyl-5*H*-[1,2,4]triazino[5,6*-b*]indol-3-yl)thio)acetic acid (5)**. The title compound was prepared from *tert*-butyl 2-((5-methyl-5*H*-[1,2,4]triazino[5,6*-b*]indol-3-yl)thio)acetate (**3**) (0.40 g, 1.21 mmol) according to general procedure B. The product was obtained as a yellow solid (280 mg, 84.34%). ^1^H NMR (400 MHz, DMSO-*d*_6_) δ 8.34 (d, *J* = 7.7 Hz, 1H), 7.79 (d, *J* = 4.0 Hz, 2H), 7.51 (dq, *J* = 8.1, 4.5 Hz, 1H), 4.10 (s, 2H), 3.83 (s, 3H). ^13^C NMR (101 MHz, DMSO-*d*_6_) δ 170.50, 166.81, 146.54, 142.14, 131.47, 123.40, 121.92, 117.75, 111.70, 79.46, 33.58, 27.81. LCMS *m/z* calc. for C_12_H_10_N_4_O_2_S [M+H]^+^: 274.4, found 274.3 with *t*_R_ 2.40 min, purity 95%.

**2-((8-Bromo-5-methyl-5*H*-[1,2,4]triazino[5,6*-b*]indol-3-yl)thio)acetic acid (6)**. The title compound was prepared from *tert*-butyl 2-((8-bromo-5-methyl-5*H*-[1,2,4]triazino[5,6*-b*]indol-3-yl)thio)acetate (**4**) (0.19 g, 0.46 mmol) according to general procedure B. The product was obtained as a yellow solid (0.13 g, 81.7%): ^1^H NMR (400 MHz, DMSO-*d*_6_) δ 8.50 (d, *J* = 2.0 Hz, 1H), 7.94 (dd, *J* = 8.7, 2.0 Hz, 1H), 7.78 (d, *J* = 8.7 Hz, 1H), 4.14 (s, 2H), 3.81 (s, 3H). 13C NMR (101 MHz, DMSO-*d*_6_) δ 168.10, 146.66, 140.93, 133.76, 124.20, 119.65, 115.51, 115.49, 113.88, 81.73, 34.30, 28.15. LCMS *m/z* calc for C_12_H_9_BrN_4_O_2_S [M+H]^+^: 353.2, found 353.2 with *t*_R_ 2.63 min, purity 95%.

### General Procedure C: HATU-Mediated Amide Bond Formation (7–28)

Carboxylic acids (**5–6**) (1.0 eq), HATU (1.5 equiv.), DMAP (0.10 equiv.) and various anilines (1.0 equiv.) were dissolved in anhydrous NMP (0.1 mmol/ 3 mL) and stirred at room temperature for 5 min before addition of DIPEA (6 equiv.). The reaction mixture was allowed to stir at room temperature for overnight. The reaction was monitored by TLC and quenched by addition of water (20 mL). The mixture was diluted with water (10 mL) and extracted with EtOAc (15 mL × 3) and the combined organic layers were washed with Sat. NaHCO3 (20 mL × 3) and brine (20 mL) and dried over Na_2_SO_4_. Solvent was removed under vacuum and the crude product was purified by column chromatography to afford the target compounds.

***N***-**(4-Chlorophenyl)-2-((5-methyl-5*H*-[1,2,4]triazino[5,6*-b*]indol-3-yl)thio)acetamide (7)**. The title compound was prepared from 2-((5-methyl-5*H*-[1,2,4]triazino[5,6*-b*]indol-3-yl)thio)acetic acid (**5**) (0.050 g, 0.18 mmol) and 4-chloroaniline (0.023 g, 0.18 mmol) according to general procedure C. The crude product was purified by column chromatography (petroleum ether/EtOAc 2:1) to give a white solid (0.022 g, 31%). ^1^H NMR (400 MHz, DMSO-*d*_6_) δ 10.56 (s, 1H), 8.32 (d, *J* = 7.9 Hz, 1H), 7.81–7.72 (m, 2H), 7.71–7.63 (m, 2H), 7.48 (td, *J* = 6.9, 5.9, 2.2 Hz, 1H), 7.42–7.33 (m, 2H), 4.28 (s, 2H), 3.77 (s, 3H). ^13^C NMR (101 MHz, DMSO-*d*_6_) δ 167.07, 166.92, 146.51, 142.05, 141.40, 138.50, 131.38, 129.19, 127.37, 123.32, 121.86, 121.11, 117.71, 111.62, 35.99, 27.72. LCMS *m/z* calc.for C_18_H_14_ClN_5_OS [M+H]^+^: 383.3, found 383.9 with *t*_R_ 2.84 min, purity 95%.

**2-((8-Bromo-5-methyl-5*H*-[1,2,4]triazino[5,6*-b*]indol-3-yl)thio)*-N-*(4-chlorophenyl)acetamide (8)**. The title compound was prepared from 2-((8-bromo-5-methyl-5*H*-[1,2,4]triazino[5,6*-b*]indol-3-yl)thio)acetic acid (**6**) (0.050 g, 0.1415 mmol) and 4-chloroaniline (0.020 g, 0.1562 mmol) according to general procedure C. The crude product was purified by column chromatography (petroleum ether/EtOAc 2:1) to give a white solid (0.010 g, 15%). ^1^H NMR (400 MHz, DMSO-*d*_6_) δ 10.57 (s, 1H), 8.50 (d, *J* = 2.0 Hz, 1H), 7.93 (dd, *J* = 8.7, 2.0 Hz, 1H), 7.76 (d, *J* = 8.7 Hz, 1H), 7.72–7.60 (m, 2H), 7.42–7.34 (m, 2H), 4.28 (s, 1H), 3.77 (s, 1H). ^13^C NMR (101 MHz, DMSO-*d*_6_) δ 167.86, 166.82, 146.71, 140.96, 140.55, 138.49, 133.71, 129.20, 127.38, 124.18, 121.11, 119.71, 115.45, 113.86, 36.01, 27.90. LCMS *m/z* calc. for C_18_H_14_BrClN_5_OS [M+H]^+^: 462.3, found 462.7 with *t*_R_ 2.91 min, purity 95%.

**2-((5-Methyl-5*H*-[1,2,4]triazino[5,6*-b*]indol-3-yl)thio)*-N-*phenylacetamide (9)**. The title compound was prepared from 2-((5-methyl-5*H*-[1,2,4]triazino[5,6*-b*]indol-3-yl)thio)acetic acid (**5**) (0.050 g, 0.18 mmol) and aniline (0.017 g, 0.18 mmol) according to general procedure C. The crude product was purified by column chromatography (petroleum ether/EtOAc 2:1) to give a white solid (0.013 g, 20%). ^1^H NMR (400 MHz, DMSO-*d*_6_) δ 10.42 (s, 1H), 8.33 (d, *J* = 7.8 Hz, 1H), 7.81–7.72 (m, 2H), 7.66–7.58 (m, 2H), 7.49 (ddd, *J* = 8.1, 5.2, 3.0 Hz, 1H), 7.32 (t, *J* = 7.9 Hz, 2H), 7.11–7.02 (m, 1H), 4.28 (s, 2H), 3.79 (s, 3H). ^13^C NMR (101 MHz, DMSO-*d*_6_) δ 167.17, 166.70, 146.54, 142.07, 141.40, 139.56, 131.39, 129.27, 123.83, 123.34, 121.88, 119.57, 117.74, 111.65, 36.01, 27.75. LCMS *m/z* calc for C_18_H_15_N_5_OS [M+H]^+^: 349.4, found 349.4 with *t*_R_ 2.72 min, purity 95%.

**2-((8-Bromo-5-methyl-5*H*-[1,2,4]triazino[5,6*-b*]indol-3-yl)thio)*-N-*phenylacetamide (10)**. The title compound was prepared from 2-((8-bromo-5-methyl-5*H*-[1,2,4]triazino[5,6*-b*]indol-3-yl)thio)acetic acid (**6**) (0.050 g, 0.14 mmol) and aniline (0.013 g, 0.14 mmol) according to general procedure C. The crude product was purified by column chromatography (petroleum ether/EtOAc 2:1) to give a white solid (0.015 g, 25%). ^1^H NMR (400 MHz, DMSO-*d*_6_) δ 10.42 (s, 1H), 8.49 (d, *J* = 1.9 Hz, 1H), 7.93 (dd, *J* = 8.7, 2.0 Hz, 1H), 7.76 (d, *J* = 8.7 Hz, 1H), 7.62 (d, *J* = 7.9 Hz, 2H), 7.32 (t, *J* = 7.9 Hz, 2H), 7.06 (t, *J* = 7.4 Hz, 1H), 4.29 (s, 2H), 3.78 (s, 3H). ^13^C NMR (101 MHz, DMSO-*d*_6_) δ 167.96, 166.60, 146.71, 140.94, 140.52, 139.54, 133.69, 132.07, 129.27, 129.14, 124.16, 123.84, 119.71, 119.57, 115.44, 113.85, 36.03, 27.91. LCMS *m/z* calc. for C_18_H_14_BrN_5_OS [M+H]^+^: 429.2, found 428.3 with *t*_R_ 2.87 min, purity 95%.

***N***-**(3-Chlorophenyl)-2-((5-methyl-5*H*-[1,2,4]triazino[5,6*-b*]indol-3-yl)thio)acetamide (11)**. The title compound was prepared from 2-((5-methyl-5*H*-[1,2,4]triazino[5,6*-b*]indol-3-yl)thio)acetic acid (**5**) (0.040 g, 0.15 mmol) and 3-chloroaniline (0.019 g, 0.15 mmol) according to general procedure C. The crude product was purified by column chromatography (petroleum ether/EtOAc 2:1) to give white solid (0.013 g, 23%). ^1^H NMR (400 MHz, DMSO-*d*_6_) δ 10.62 (s, 1H), 8.34 (d, *J* = 7.8 Hz, 1H), 7.84 (t, *J* = 2.0 Hz, 1H), 7.78 (d, *J* = 3.9 Hz, 2H), 7.54–7.28 (m, 2H), 7.36 (t, *J* = 8.1 Hz, 1H), 7.18–7.05 (m, 1H), 4.29 (s, 2H), 3.78 (s, 3H). ^13^C NMR (101 MHz, DMSO-*d*_6_) δ 167.19, 167.01, 146.55, 142.11, 141.46, 140.98, 133.60, 131.42, 131.02, 123.56, 123.35, 121.91, 119.00, 117.94, 117.74, 111.67, 36.01, 27.74. LCMS *m/z* calc.for C_15_H_14_ClN_5_OS [M+H]^+^: 383.8, found 383.9 with *t*_R_ 2.87 min, purity 95%.

**2-((8-Bromo-5-methyl-5*H*-[1,2,4]triazino[5,6*-b*]indol-3-yl)thio)*-N-*(3-chlorophenyl)acetamide (12)**. The title compound was prepared from 2-((8-bromo-5-methyl-5*H*-[1,2,4]triazino[5,6*-b*]indol-3-yl)thio)acetic acid (**6**) (0.050 g, 0.14 mmol) and 3-chloroaniline (0.018 g, 0.14 mmol) according to general procedure C. The crude product was purified by column chromatography (petroleum ether/EtOAc 2:1) to give a white solid (0.010 g, 15.4%). ^1^H NMR (400 MHz, DMSO-*d*_6_) δ 10.63 (s, 1H), 8.50 (d, *J* = 1.9 Hz, 1H), 7.93 (dd, *J* = 8.7, 2.0 Hz, 1H), 7.84 (t, *J* = 2.1 Hz, 1H), 7.76 (d, *J* = 8.6 Hz, 1H), 7.52–7.45 (m, 1H), 7.35 (d, *J* = 8.1 Hz, 1H), 7.16–7.07 (m, 1H), 4.29 (s, 2H), 3.77 (s, 3H). ^13^C NMR (101 MHz, DMSO-*d*_6_) δ 167.79, 167.09, 146.71, 140.96, 140.58, 133.71, 133.60, 131.02, 124.17, 123.57, 119.69, 119.00, 117.94, 115.45, 113.86, 36.03, 27.90. LCMS *m/z* calc. for C_18_H_14_BrClN_5_O_S_ [M+H]^+^: 464.2, found 464.3 with *t*_R_ 2.98 min, purity 95%.

***N***-**(3,4-Dichlorophenyl)-2-((5-methyl-5*H*-[1,2,4]triazino[5,6*-b*]indol-3-yl)thio)acetamide (13)**. The title compound was prepared from 2-((5-methyl-5*H*-[1,2,4]triazino[5,6*-b*]indol-3-yl)thio)acetic acid (**5**) (0.070 g, 0.25 mmol) and 3,4-dichloroaniline (0.041 g, 0.25 mmol) according to general procedure C. The crude product was purified by column chromatography (petroleum ether/EtOAc 2:1) to give a white solid (0.005 g, 5%). ^1^H NMR (400 MHz, DMSO-*d*_6_) δ 10.73 (s, 1H), 8.33 (d, *J* = 7.8 Hz, 1H), 8.02 (d, *J* = 2.4 Hz, 1H), 7.84–7.74 (m, 2H), 7.60 (d, *J* = 8.8 Hz, 1H), 7.56–7.28 (m, 2H), 4.29 (s, 2H), 3.78 (s, 3H). ^13^C NMR (101 MHz, DMSO-*d*_6_) δ 167.37, 166.94, 146.55, 142.12, 141.48, 139.62, 131.53, 131.44, 131.28, 125.26, 123.37, 121.91, 120.72, 119.61, 117.74, 111.69, 36.00, 27.75. LCMS *m/z* calc.for C_18_H_13_Cl_2_N_5_OS [M+H]^+^: 418.3, found 418.2 with *t*_R_ 2.95 min, purity 95%.

**2-((8-Bromo-5-methyl-5*H*-[1,2,4]triazino[5,6*-b*]indol-3-yl)thio)*-N-*(3,4-dichlorophenyl)acetamide (14)**. The title compound was prepared from 2-((8-bromo-5-methyl-5*H*-[1,2,4]triazino[5,6*-b*]indol-3-yl)thio)acetic acid (**6**) (0.070 g, 0. 20 mmol) and 3,4-dichloroaniline (0.035 g, 0.21 mmol) according to general procedure C. The crude product was purified by column chromatography (petroleum ether/EtOAc 2:1) to give white solid (0.005 g, 5%). ^1^H NMR (400 MHz, DMSO-*d*_6_) δ 10.73 (d, *J* = 7.6 Hz, 1H), 8.50 (d, *J* = 1.9 Hz, 1H), 8.02 (d, *J* = 2.3 Hz, 1H), 7.94 (dd, *J* = 8.7, 1.9 Hz, 1H), 7.76 (d, *J* = 8.7 Hz, 1H), 7.60 (d, *J* = 8.8 Hz, 1H), 7.53 (dd, *J* = 8.9, 2.4 Hz, 1H), 4.29 (s, 2H), 3.76 (s, 3H). ^13^C NMR (101 MHz, DMSO-*d*_6_) δ 167.37, 166.94, 146.55, 142.12, 141.48, 139.62, 131.53, 131.44, 131.28, 125.26, 123.37, 121.91, 120.72, 119.61, 117.74, 111.69, 36.00, 27.75. LCMS *m/z* calc. for C_18_H_12_BrCl_2_N_5_OS [M+H]^+^: 497.2, found 497.19 with *t*_R_ 3.06 min, purity 95%.

***N***-**(4-Fluorophenyl)-2-((5-methyl-5*H*-[1,2,4]triazino[5,6*-b*]indol-3-yl)thio)acetamide (15)**. The title compound was prepared from 2-((5-methyl-5*H*-[1,2,4]triazino[5,6*-b*]indol-3-yl)thio)acetic acid (**5**) (0.070 g, 0.26 mmol) and 4-fluoroaniline (0.028 g, 0.26 mmol) according to general procedure C. The crude product was purified by column chromatography (petroleum ether/EtOAc 2:1) to give a white solid (0.030 g, 32%). ^1^H NMR (400 MHz, DMSO-*d*_6_) δ 10.48 (s, 1H), 8.33 (d, *J* = 7.8 Hz, 1H), 7.86–7.71 (m, 2H), 7.69–7.59 (m, 2H), 7.49 (ddd, *J* = 8.0, 5.3, 2.9 Hz, 1H), 7.16 (t, *J* = 8.9 Hz, 2H), 4.27 (s, 2H), 3.78 (s, 3H). ^13^C NMR (101 MHz, DMSO-*d*_6_) δ 167.12, 166.64, 146.54, 142.08, 141.41, 135.96, 131.40, 123.34, 121.88, 121.38, 121.30, 117.74, 115.96, 115.74, 111.65, 35.91, 27.74. LCMS *m/z* calc.for C_18_H_14_FN_5_OS [M+H]^+^: 367.1, found 367.4 with *t*_R_ 2.75 min, purity 95%.

**2-((8-Bromo-5-methyl-5*H*-[1,2,4]triazino[5,6*-b*]indol-3-yl)thio)*-N-*(4-fluorophenyl)acetamide (16)**. The title compound was prepared from 2-((8-bromo-5-methyl-5*H*-[1,2,4]triazino[5,6*-b*]indol-3-yl)thio)acetic acid (**6**) (0.070 g, 0.20 mmol) and 4-fluoroaniline (0.022 g, 0.20 mmol) according to general procedure C. The crude product was purified by column chromatography (petroleum ether/EtOAc 2:1) to give a white solid (0.027 g, 30%): ^1^H NMR (400 MHz, DMSO-*d*_6_) δ 10.48 (s, 1H), 8.49 (d, *J* = 2.0 Hz, 1H), 7.92 (dd, *J* = 8.7, 2.0 Hz, 1H), 7.75 (d, *J* = 8.7 Hz, 1H), 7.68–7.57 (m, 2H), 7.16 (t, *J* = 8.9 Hz, 2H), 4.27 (s, 2H), 3.77 (s, 3H). ^13^C NMR (101 MHz, DMSO-*d*_6_) δ 167.91, 166.55, 146.71, 140.95, 140.54, 135.92, 133.70, 124.17, 121.38, 121.30, 119.70, 115.96, 115.74, 115.45, 113.85, 35.93, 27.90. LCMS *m/z* calc. for C_18_H_13_BrFN_5_OS [M+H]^+^: 446.3, found 446.3 with *t*_R_ 2.88 min, purity 95%.

**2-((5-Methyl-5*H*-[1,2,4]triazino[5,6*-b*]indol-3-yl)thio)*-N-*(p-tolyl)acetamide (17)**. The title compound was prepared from 2-((5-methyl-5*H*-[1,2,4]triazino[5,6*-b*]indol-3-yl)thio)acetic acid (**5**) (0.070 g, 0.26 mmol) and 4-methylaniline (0.027 g, 0.26 mmol) according to general procedure C. The crude product was purified by column chromatography (petroleum ether/EtOAc 2:1) to give a white solid (0.032 g, 35%). ^1^H NMR (400 MHz, DMSO-*d*_6_) δ 10.33 (s, 1H), 8.29 (d, *J* = 7.8 Hz, 1H), 7.74 (d, *J* = 6.8 Hz, 2H), 7.49 (dd, *J* = 17.2, 7.7 Hz, 3H), 7.12 (d, *J* = 8.0 Hz, 2H), 4.26 (s, 2H), 3.76 (s, 3H), 2.25 (s, 3H). ^13^C NMR (101 MHz, DMSO-*d*_6_) δ 167.19, 166.28, 146.48, 142.00, 141.33, 137.06, 132.74, 131.33, 129.63, 123.29, 121.82, 119.58, 117.70, 111.57, 35.98, 27.72, 20.91. LCMS *m/z* calc. for C_19_H_17_N_5_OS [M+H]^+^: 363.4, found 363.4 with *t*_R_ 2.78 min, purity 95%.

**2-((8-Bromo-5-methyl-5*H*-[1,2,4]triazino[5,6*-b*]indol-3-yl)thio)*-N-*(p-tolyl)acetamide (18)**. The title compound was prepared from 2-((8-bromo-5-methyl-5*H*-[1,2,4]triazino[5,6*-b*]indol-3-yl)thio)acetic acid (**6**) (0.070 g, 0.20 mmol) and 4-methylaniline (0.030 g, 0.20 mmol) according to general procedure C. The crude product was purified by column chromatography (petroleum ether/EtOAc 2:1) to give a white solid (0.015 g, 12 %). ^1^H NMR (400 MHz, DMSO-*d*_6_) δ 10.34 (s, 1H), 8.47 (d, *J* = 2.0 Hz, 1H), 7.91 (dd, *J* = 8.7, 2.0 Hz, 1H), 7.74 (d, *J* = 8.7 Hz, 1H), 7.51 (d, *J* = 8.4 Hz, 2H), 7.12 (d, *J* = 8.1 Hz, 2H), 4.27 (s, 2H), 3.77 (s, 3H), 2.25 (s, 3H). ^13^C NMR (101 MHz, DMSO-*d*_6_) δ 168.00, 166.33, 146.69, 140.91, 140.49, 137.05, 133.67, 132.74, 129.63, 124.14, 119.70, 119.58, 115.44, 113.84, 36.00, 27.91, 20.92. LCMS *m/z* calc. for C_19_H_16_BrN_5_OS [M+H]^+^: 442.3, found 442.1 with *t*_R_ 2.95 min, purity 95%.

***N***-**(4-Methoxyphenyl)-2-((5-methyl-5*H*-[1,2,4]triazino[5,6-*b*]indol-3-yl)thio)acetamide (19)**. The title compound was prepared from 2-((5-methyl-5*H*-[1,2,4]triazino[5,6*-b*]indol-3-yl)thio)acetic acid (**5**) (0.070 g, 0.26 mmol) and 4-methoxy aniline (0.031 g, 0.26 mmol) according to general procedure C. The product was obtained as a yellow solid (0.095 g, 98%). 1H NMR (400 MHz, DMSO-d6) δ 10.27 (s, 1H, H-7), 8.32 (d, *J* = 7.7 Hz, 1H), 7.88–7.70 (m, 2H), 7.56–7.51 (m, 2H), 7.48 (ddd, *J* = 2.6, 5.6, 8.1 Hz, 1H), 6.96–6.71 (m, 2H), 4.25 (s, 2H, H-6), 3.79 (s, 3H), 3.72 (s, 3H). 13C NMR (101 MHz, DMSO-d6) δ 167.20, 166.15, 155.75, 146.45, 142.06, 141.37, 132.70, 131.38, 123.33, 121.86, 121.13, 117.75, 114.37, 111.64, 38.71, 35.88, 27.74. LCMS m/z calc for C19H17N5O2S+ [M+H]^+^: 380.4, found 379.4 with tR 2.70 min, purity 95%.

**2-((8-Bromo-5-methyl-5*H*-[1,2,4]triazino[5,6*-b*]indol-3-yl)thio)*-N-*(4-methoxyphenyl)acetamide (20)**. The title compound was prepared from 2-((8-bromo-5-methyl-5*H*-[1,2,4]triazino[5,6*-b*]indol-3-yl)thio)acetic acid (**6**) (0.070 g, 0.20 mmol) and 4-methoxyaniline (0.023 g, 0.20 mmol) according to general procedure C. The crude product was purified by column chromatography (petroleum ether/EtOAc 2:1) to give a light yellow solid (0.017 g, 18%). ^1^H NMR (400 MHz, DMSO-*d*_6_) δ 10.28 (s, 1H), 8.49 (d, *J* = 2.0 Hz, 1H), 7.93 (dd, *J* = 8.7, 2.0 Hz, 1H), 7.76 (d, *J* = 8.7 Hz, 1H), 7.56–7.49 (m, 2H), 6.93–6.85 (m, 2H), 4.25 (s, 2H, H5), 3.78 (s, 3H), 3.72 (s, 3H). ^13^C NMR (101 MHz, DMSO-*d*_6_) δ 168.03, 166.05, 155.76, 146.72, 140.94, 140.50, 133.68, 132.69, 124.15, 121.13, 119.72, 115.44, 114.38, 113.85, 38.72, 35.90, 27.92. LCMS *m/z* calc. for C_19_H_17_BrN_5_O_2_S [M+H]^+^: 458.3, found 458.3 with *t*_R_ 2.87 min, purity 95%.

**2-((5-Methyl-5*H*-[1,2,4]triazino[5,6*-b*]indol-3-yl)thio)*-N-*(4(trifluoromethoxy)phenyl)acetamide (21)**. The title compound was prepared from 2-((5-methyl-5*H*-[1,2,4]triazino[5,6*-b*]indol-3-yl)thio)acetic acid (**5**) (0.050 g, 0.18 mmol) and 4-trifluoromethoxyaniline (0.032 g, 0.18 mmol) according to general procedure C. The crude product was purified by column chromatography (petroleum ether/EtOAc 2:1) to give a white solid (0.005 g, 6%). ^1^H NMR (400 MHz, DMSO-*d*_6_) δ 10.63 (s, 1H), 8.33 (d, *J* = 7.9 Hz, 1H), 7.84–7.62 (m, 4H), 7.49 (ddd, *J* = 8.1, 5.3, 2.8 Hz, 1H), 7.34 (d, *J* = 8.6 Hz, 2H), 4.29 (s, 2H), 3.78 (d, *J* = 1.7 Hz, 3H). ^13^C NMR (101 MHz, DMSO-*d*_6_) δ 178.30, 167.05, 167.00, 146.55, 142.10, 141.44, 138.74, 131.41, 123.35, 122.18, 121.90, 120.92, 117.74, 111.66, 35.95, 27.75. LCMS *m/z* calc. for C_19_H_14_F_3_N_5_O_2_S [M+H]^+^: 283.4, found 283.4 with *t*_R_ 2.91 min, purity 95%.

**2-((8-Bromo-5-methyl-5*H*-[1,2,4]triazino[5,6*-b*]indol-3-yl)thio)*-N-*(4- (trifluoromethoxy)phenyl)acetamide (22)**. The title compound was prepared from 2-((8-bromo-5-methyl-5*H*-[1,2,4]triazino[5,6*-b*]indol-3-yl)thio)acetic acid (**6**) (0.050 g, 0.14 mmol) and 4-trifluoromethoxyaniline (0.021 mL, 0.16 mmol) according to general procedure C. The crude product was purified by column chromatography (petroleum ether/EtOAc 2:1) to give a white solid (0.007 g, 9%). ^1^H NMR (400 MHz, DMSO-*d*_6_) δ 10.63 (s, 1H), 8.48 (d, *J* = 2.0 Hz, 1H), 7.92 (dd, *J* = 8.7, 2.0 Hz, 1H), 7.83–7.64 (m, 3H), 7.34 (d, *J* = 8.6 Hz, 2H), 4.29 (s, 2H), 3.77 (d, *J* = 5.3 Hz, 3H). ^13^C NMR (101 MHz, DMSO-*d*_6_) δ 167.83, 167.00, 166.91, 146.70, 142.09, 140.95, 140.56, 138.73, 133.70, 124.16, 122.18, 120.93, 119.69, 119.33, 115.45, 113.85, 111.66, 35.97, 27.74. LCMS *m/z* calc. for C_19_H_13_BrF_3_N_5_O_2_S [M+H]^+^: 512.1, found 512.3 with *t*_R_ 3.05 min, purity 95%.

***N***-**(4-(Cyanomethyl)phenyl)-2-((5-methyl-5*H*-[1,2,4]triazino[5,6*-b*]indol-3-yl)thio)acetamide (23)**. The title compound was prepared from 2-((5-methyl-5*H*-[1,2,4]triazino[5,6*-b*]indol-3-yl)thio)acetic acid (**5**) (0.080 g, 0.0.29 mmol) and 2-(4-aminophenyl)acetonitrile (0.028 g, 0.32 mmol) according to general procedure C. The crude product was purified by column chromatography (petroleum ether/EtOAc 2:1) to give a white solid (58.9 mg, 52%). ^1^H NMR (400 MHz, DMSO-*d*_6_) δ 10.52 (d, *J* = 10.1 Hz, 1H), 8.29 (d, *J* = 7.7 Hz, 1H), 7.95 (s, 1H), 7.78–7.68 (m, 2H), 7.67–7.60 (m, H), 7.46 (ddd, *J* = 1.8, 6.5, 8.0 Hz, 1H), 7.30 (d, *J* = 8.3 Hz, 2H), 4.27 (s, 2H), 3.97 (s, 2H), 3.76 (s, 3H). ^13^C NMR (101 MHz, DMSO-*d*_6_) δ 167.12, 166.85, 146.49, 142.01, 141.34, 138.94, 131.39, 129.04, 126.39, 123.33, 121.84, 120.00, 119.81, 117.67, 111.58, 35.96, 27.70, 22.30. LCMS *m/z* calc. for C_20_H_17_N_6_OS [M+H]^+^: 389.4, found 389.3 with *t*_R_ 2.66 min, purity > 95%.

**2-((5-Methyl-5*H*-[1,2,4]triazino[5,6*-b*]indol-3-yl)thio)*-N-*(4-phenoxyphenyl)acetamide (24)**. The title compound was prepared from 2-((5-methyl-5*H*-[1,2,4]triazino[5,6*-b*]indol-3-yl)thio)acetic acid (**5**) (0.10 g, 0.36 mmol) and 4-phenoxyaniline (0.067 g, 0.36 mmol) according to general procedure C. The crude product was purified by column chromatography (petroleum ether/EtOAc 2:1) to give a white solid (0.094 g, 58%). ^1^H NMR (400 MHz, DMSO-*d*_6_) δ 10.45 (s, 1H), 8.35–8.27 (m, 1H), 7.82–7.71 (m, 2H), 7.70–7.60 (m, 2H), 7.48 (ddd, *J* = 8.1, 5.6, 2.6 Hz, 1H), 7.40–7.31 (m, 2H), 7.13–7.05 (m, 1H), 7.04–6.92 (m, 4H), 4.28 (s, 2H), 3.79 (s, 3H). ^13^C NMR (101 MHz, DMSO-*d*_6_) δ 174.23, 166.53, 142.06, 141.39, 135.47, 131.38, 130.28, 123.46, 123.32, 121.86, 121.29, 119.98, 118.34, 117.74, 111.63, 35.93, 27.75. LCMS *m/z* calc. for C_24_H_19_N_5_O_2_S [M+H]^+^: 441.5, found 441.5 with *t*_R_ 2.95 min, purity 95%.

**2-((8-Bromo-5-methyl-5*H*-[1,2,4]triazino[5,6*-b*]indol-3-yl)thio)*-N-*(4-phenoxyphenyl)acetamide (25)**. The title compound was prepared from 2-((8-bromo-5-methyl-5*H*-[1,2,4]triazino[5,6*-b*]indol-3-yl)thio)acetic acid (**6**) (0.10 g, 0.28 mmol) and 4-phenoxy aniline (0.052 g, 0.28 mmol) according to general procedure C. The crude product was purified by column chromatography (petroleum ether/EtOAc 2:1) to give a white solid (0.027 g, 18%). ^1^H NMR (400 MHz, DMSO-*d*_6_) δ 10.46 (s, 1H), 8.47 (d, *J* = 2.0 Hz, 1H), 7.91 (dd, *J* = 8.7, 2.0 Hz, 1H), 7.74 (d, *J* = 8.7 Hz, 1H), 7.68–7.58 (m, 2H), 7.42–7.29 (m, 2H), 7.13–7.07 (m, 1H), 7.05–6.90 (m, 4H), 4.28 (s, 2H), 3.78 (s, 3H). ^13^C NMR (101 MHz, DMSO-*d*_6_) δ 166.28, 157.78, 152.30, 146.68, 140.92, 140.51, 135.45, 133.67, 130.28, 124.13, 123.47, 121.29, 119.97, 119.68, 118.35, 115.44, 113.83, 35.96, 27.92 LCMS *m/z* calc. for C_24_H_18_BrN_5_O_2_S+ [M+H]^+^: 520.4, found 520.4 with *t*_R_ 3.06 min, purity 95%.

***N***-**(4-Bromophenyl)-2-((5-methyl-5*H*-[1,2,4]triazino[5,6*-b*]indol-3-yl)thio)acetamide (26)**. The title compound was prepared from 2-((5-methyl-5*H*-[1,2,4]triazino[5,6*-b*]indol-3-yl)thio)acetic acid (**5**) (0.070 g, 0.26 mmol) and 4-bromoaniline (0.044 g, 0.26 mmol) according to general procedure C. The crude product was purified by column chromatography (petroleum ether/EtOAc 2:1) to give a white solid (0.030 g, 27%). ^1^H NMR (400 MHz, DMSO-*d*_6_) δ 10.56 (s, 1H), 8.33 (d, *J* = 7.7 Hz, 1H), 7.89–7.72 (m, 2H), 7.69–7.57 (m, 2H), 7.55–7.28 (m, 3H), 4.28 (s, 2H), 3.77 (s, 3H). ^13^C NMR (101 MHz, DMSO-*d*_6_) δ 167.08, 166.94, 146.55, 142.10, 141.28, 138.92, 132.11, 131.42, 123.36, 121.90, 121.50, 117.75, 115.40, 111.68, 36.01, 27.75. LCMS *m/z* calc. for C_18_H_14_BrN_5_OS [M+H]^+^: 428.3, found 428.3 with *t*_R_ 2.87 min, purity 95%.

**2-((8-Bromo-5-methyl-5*H*-[1,2,4]triazino[5,6*-b*]indol-3-yl)thio)*-N-*(4-bromophenyl)acetamide (27)**. The title compound was prepared from 2-((8-bromo-5-methyl-5*H*-[1,2,4]triazino[5,6*-b*]indol-3-yl)thio)acetic acid (**6**) (0.070 g, 0.20 mmol) and 4-bromoaniline (0.034 g, 0.20 mmol) according to general procedure C. The crude product was purified by column chromatography (petroleum ether/EtOAc 2:1) to give a yellow solid (0.007 g, 7%). ^1^H NMR (400 MHz, DMSO-*d*_6_) δ 10.57 (s, 1H), 8.49 (d, *J* = 2.0 Hz, 1H), 7.93 (dd, *J* = 8.7, 2.1 Hz, 1H), 7.76 (d, *J* = 8.7 Hz, 1H), 7.64–7.56 (m, 2H), 7.55–7.44 (m, 2H), 4.28 (s, 2H), 3.76 (s, 3H). ^13^C NMR (101 MHz, DMSO-*d*_6_) δ 167.08, 166.95, 146.55, 142.10, 141.44, 138.92, 132.11, 131.42, 123.36, 121.91, 121.50, 117.75, 115.39, 111.68, 36.01, 27.75. LCMS *m/z* calc. for C_18_H_13_Br_2_N_5_OS [M+H]^+^: 507.2, found 507.2 with *t*_R_ 2.87 min, purity 95%.

**2-((5-Methyl-5*H*-[1,2,4]triazino[5,6*-b*]indol-3-yl)thio)*-N-*(4-(pyrimidin-2yloxy)phenyl)acetamide (28)**. 4-(pyrimidin-2-yloxy)aniline (0.065 g, 0.238 mmol), 2-((5-methyl-5*H*-[1,2,4]triazino[5,6*-b*]indol-3-yl)thio)acetic acid (**5**) (0.064 g, 0.238 mmol), HATU (0.135 g, 0.357 mmol), DIPEA (0.165 mL, 0.952 mmol), and DMAP (0.030 g, 0.0238 mmol) were dissolved in NMP (2 mL). The reaction mixture was allowed to stir at room temperature overnight and monitored by TLC. The crude product was purified by column chromatography (petroleum ether/EtOAc 1:1) to give a white solid (31 mg, 29%). ^1^H NMR (400 MHz, DMSO-*d*_6_) δ 10.50 (s, 1H), 8.63 (d, *J* = 4.8 Hz, 2H), 8.31 (d, *J* = 7.8 Hz, 1H), 7.75 (d, *J* = 5.2 Hz, 2H), 7.67 (d, *J* = 8.5 Hz, 2H), 7.47 (ddd, *J* = 2.6, 5.8, 8.2 Hz, 1H), 7.25 (t, *J* = 4.8 Hz, 1H), 7.16 (d, *J* = 8.4 Hz, 2H), 4.30 (s, 2H), 3.79 (s, 3H). ^13^C NMR (101 MHz, DMSO-*d*_6_) δ 167.13, 166.67, 165.37, 160.47, 148.77, 146.53, 142.06, 141.40, 136.67, 131.38, 123.32, 122.46, 121.88, 120.83, 117.73, 117.30, 111.61, 35.96, 27.76. LCMS *m/z* calc. for C_22_H_18_N_7_O_2_S^+^ [M+H]^+^: 444.5, found 444.3 with *t*_R_ 2.62 min, purity >95%.

### General Procedure D: Acylation of Anilines to Form Bromoacetatamides (32–37)

Aniline, 4-chloroaniline or 4-chloro*-N-*methylaniline and Et_3_N (2 equiv.) were dissolved in anhydrous DCM at 0°C under N_2_ protection followed by addition of various bromoacetyl chlorides (**29–31**) (1.0 equiv.). The reaction mixture was allowed to slowly warm up to room temperature and stirred for 4 h with monitored by TLC. Once the reaction was complete, solvent was removed under vacuum and crude product was used directly for next step without further purification (Deora et al., 2019).

**2-Bromo*-N-*(4-(pyridin-2-yloxy)phenyl)acetamide (33)**. The title compound was prepared according to general procedures B from 4-(Pyridin-2-yloxy) aniline (100 mg, 32.57 mmol), Et_3_N (3 equiv.) and 2-bromoacetyl chloride (**29**) (1.1 equiv.). The crude product was purified by column chromatography (petroleum ether/EtOAc 5:1) to give a solid (71 mg, 47%). ^1^H NMR (400 MHz, CDCl_3_) δ 8.19 (dd, *J* = 2.0, 5.1 Hz, 1H), 7.64 (ddd, *J* = 2.0, 7.1, 8.2 Hz, 1H), 6.99–6.90 (m, 3H), 6.83 (dd, *J* = 1.0, 8.3 Hz, 1H), 6.76–6.67 (m, 2H), 3.65 (s, 2H). ^13^C NMR (101 MHz, CDCl_3_) δ 164.54, 147.74, 146.04, 143.50, 139.19, 122.29, 117.88, 116.14, 110.78, 45.81. LCMS *m/z* calc. for C_13_H_11_BrN_2_O_2_ [M+H]^+^: 307.1, found 307.0 with *t*_R_ 2.59 min, purity > 95%.

**2-Bromo*-N-*(4-chlorophenyl)acetamide (37)**. The title compound was prepared from 2-bromopropanoyl chloride (**30**) (0.10 g, 0.71 mmol) and 4-chloro*-N-*methylaniline (0.12 g, 0.71 mmol) according to general procedure D. The crude product was purified by column chromatography (petroleum ether/EtOAc 6:1) to give a white solid (149 mg, 76%). ^1^H NMR (400 MHz, CDCl_3_) δ 7.44 (d, *J* = 8.3 Hz, 2H), 7.26 (d, *J* = 8.2 Hz, 2H), 4.24 (q, *J* = 6.6 Hz, 1H), 3.28 (s, 3H), 1.74 (d, *J* = 6.7 Hz, 3H). ^13^C NMR (101 MHz, CDCl_3_) δ 169.47, 141.35, 134.42, 130.22, 128.64, 38.78, 38.13, 21.74. LCMS *m/z* calc. for C_8_H_8_BrClNO [M+H]^+^: 249.5, found 250.0 with *t*_R_ 2.63 min, purity > 95%.

### General Procedure E: Alkylation of Thiols (1–2) With Bromoacetamides (32–37) to Give Thioethers (38–44)

The substituted 5-methyl-5*H*-[1,2,4]triazino[5,6*-b*]indole-3-thiol intermediates **1–2** (1 equiv.) and Et_3_N (2 equiv.) were dissolved in anhydrous DCM (0.1 mmol/5 mL) followed by addition of bromoacetamides (**32–37**) (1.0 equiv.) at 0°C under N_2_. The reaction mixture was allowed slowly warm up to room temperature and stir for 4 h. The reaction was monitored by TLC. Once the reaction finished, solvent was removed under vacuum. The crude product was purified by column chromatography.

***N***-**(2-Chlorophenyl)-2-((5-methyl-5*H*-[1,2,4]triazino[5,6*-b*]indol-3-yl)thio)acetamide (38)**. The title compound was prepared from 2-bromo*-N-*(2-chlorophenyl)acetamide (**32**) (Sun et al., 2014) (0.1 g, 0.41 mmol) and 5-methyl-5*H*-[1,2,4]triazino[5,6*-b*]indole-3-thiol (**1**) (0.088 g, 0.41 mmol) according to general procedure E. The crude product was purified by column chromatography (petroleum ether/EtOAc 2:1) to give a white solid (0.029 g, 18%). ^1^H NMR (400 MHz, DMSO-*d*_6_) δ 9.93 (s, 1H), 8.34 (d, *J* = 7.7 Hz, 1H), 7.87–7.73 (m, 3H), 7.50 (ddd, *J* = 7.7, 5.1, 2.4 Hz, 2H), 7.40–7.25 (m, 1H), 7.18 (td, *J* = 7.7, 1.6 Hz), 4.35 (s, 2H), 3.81 (s, 3H). ^13^C NMR (101 MHz, DMSO-*d*_6_) δ 167.33, 166.90, 146.55, 142.14, 141.50, 135.29, 131.47, 129.94, 127.99, 126.66, 125.83, 123.39, 121.93, 117.74, 111.68, 35.28, 27.85. LCMS *m/z* calc. for C_18_H_15_ClN_5_OS [M+H]^+^: 384.2, found 383.8 with *t*_R_ 2.82 min, purity 95%.

**2-((8-Bromo-5-methyl-5*H*-[1,2,4]triazino[5,6*-b*]indol-3-yl)thio)*-N-*(2-chlorophenyl)acetamide (39)**. The title compound was prepared from 2-bromo*-N-*(2-chlorophenyl)acetamide (**32**) (0.1 g, 0.3387 mmol) and 8-bromo-5-methyl-5*H*[1,2,4]triazino[5,6*-b*]indole-3-thiol (**2**) (0.083 g, 0.3387 mmol) according to general procedure E. The crude product was purified by column chromatography (petroleum ether/EtOAc 2:1) to give a white solid (0.053 g, 35%). ^1^H NMR (400 MHz, DMSO-*d*_6_) δ 9.94 (s, 1H), 8.50 (t, *J* = 2.8 Hz, 1H), 7.93 (dt, *J* = 9.3, 2.7 Hz, 1H), 7.88–7.64 (m, 2H), 7.50 (dd, *J* = 7.9, 1.6 Hz, 1H), 7.28–7.25 (m, 1H), 7.23–7.08 (m, 1H), 4.36 (d, *J* = 3.3 Hz, 2H), 3.80 (s, 3H). ^13^C NMR (101 MHz, DMSO-*d*_6_) δ 167.71, 167.22, 146.72, 140.99, 140.61, 135.28, 133.76, 129.96, 127.98, 126.70, 126.29, 125.94, 124.20, 119.69, 115.50, 113.88, 35.45, 28.00. LCMS *m/z* calc. for C_18_H_13_BrClN_5_OS [M+H]^+^: 462.7, found 462.7 with *t*_R_ 3.01 min, purity 95%.

**2-((5-Methyl-5*H*-[1,2,4]triazino[5,6*-b*]indol-3-yl)thio)*-N-*(4-(pyridin-2-yloxy)phenyl)acetamide (40)**. The title compound was prepared from 5-Methyl-5*H*-[1,2,4]triazino[5,6*-b*]indole-3-thiol (**1**) (0.030 g, 0.14 mmol), Et_3_N (1.5 equiv.) and 2-bromo*-N-*(4-(pyridin-2-yloxy)phenyl)acetamide (**33**) (0.051 g, 0.17 mmol) according to general procedure E. The reaction mixture was allowed slowly warm up to room temperature and stirred overnight and monitored by TLC. The crude product was purified by column chromatography (petroleum ether/EtOAc 1:1) to give a white solid (39 mg, 64%). ^1^H NMR (400 MHz, CDCl_3_) δ 9.87 (s, 1H), 8.46 (dt, *J* = 1.1, 7.8 Hz, 1H), 8.16 (ddd, *J* = 0.8, 2.1, 5.0 Hz, 1H), 7.77 (ddd, *J* = 1.2, 7.4, 8.5 Hz, 1H), 7.68–7.56 (m, 3H), 7.55–7.47 (m, 2H), 7.14–7.04 (m, 2H), 6.96 (ddd, *J* = 1.0, 5.0, 7.2 Hz, 1H), 6.86 (dt, *J* = 0.9, 8.3 Hz, 1H), 4.13 (d, *J* = 1.5 Hz, 2H), 3.87 (s, 3H). ^13^C NMR (101 MHz, CDCl_3_) δ 167.21, 167.08, 150.16, 147.70, 146.55, 142.15, 139.35, 134.91, 131.51, 123.44, 122.66, 121.76, 121.07, 118.33, 117.96, 111.22, 110.16, 35.75, 27.53. LCMS *m/z* calc. for C_23_H_19_N_6_O_2_S [M+H]^+^: 443.5, found 443.2 with *t*_R_ 2.78 min, purity > 95%.

***N***-**(4-Chlorophenyl)-2-((5-methyl-5*H*-[1,2,4]triazino[5,6-*b*]indol-3-yl)thio)propanamide (41)**. The title compound (**41**) was prepared from (**34**) (Deora et al., 2019) (0.20 g, 0.93 mmol) and (**1**) (0.24 g, 0.93 mmol) according to the general procedure E. The crude product was purified by column chromatography (petroleum ether/EtOAc 2:1) to give a white solid (0.083 g, 22%). ^1^H NMR (400 MHz, DMSO-*d*6) δ 10.61 (s, 1H), 8.30 (d, *J* = 7.7 Hz, 1H), 7.75 (d, *J* = 5.9 Hz, 2H), 7.68 (d, *J* = 8.6 Hz, 2H), 7.47 (ddd, *J* = 8.0, 6.0, 2.3 Hz, 1H), 7.38 (d, *J* = 8.8 Hz, 2H), 4.85 (q, *J* = 7.0 Hz, 1H), 3.77 (s, 3H), 1.68 (d, *J* = 7.1 Hz, 3H). ^13^C NMR (101 MHz, DMSO-*d*_6_) δ 170.55, 166.85, 146.49, 142.08, 141.47, 138.40, 131.42, 129.19, 127.53, 123.33, 121.88, 121.25, 117.71, 111.61, 45.12, 27.76, 18.65. LCMS *m/z* calc. for C_19_H_17_ClN_5_OS^+^ [M]^+^: 398.8, found 397.8 with *t*_R_ 3.03 min, purity 95%.

***N***-**(4-Chlorophenyl)*-N-*methyl-2-((5-methyl-5*H*-[1,2,4]triazino[5,6*-b*]indol-3-yl)thio)acetamide (43)**. The title compound was prepared from 2-bromo*-N-*(4-chlorophenyl)*-N-*methylacetamide (**36**) (0.050 g, 0.19 mmol) and 5-methyl-5*H*-[1,2,4]triazino[5,6*-b*]indole-3-thiol (**1**) (0.041 g, 0.19 mmol) according to general procedure E. The crude product was purified by column chromatography (petroleum ether/EtOAc 1:1) to give a white solid (32.5 mg, 28%): ^1^H NMR (400 MHz, DMSO-*d*_6_) δ 8.32 (d, *J* = 7.8 Hz, 1H), 7.78 (d, *J* = 4.1 Hz, 2H), 7.65–7.35 (m, 5H), 4.05 (d, *J* = 22.3 Hz, 2H), 3.76 (s, 3H), 3.23 (s, 3H). ^13^C NMR (101 MHz, DMSO-*d*_6_) δ 170.55, 166.85, 146.49, 142.08, 141.47, 138.40, 131.42, 129.19, 127.53, 123.33, 121.88, 121.25, 117.71, 111.61, 45.12, 27.76, 18.65. LCMS *m/z* calc. for C_19_H_17_ClN_5_OS [M+H]^+^: 398.2, found 397.8 with *t*_R_ 3.03 min, purity 95%.

***N***-**(4-Chlorophenyl)*-N-*methyl-2-((5-methyl-5*H*-[1,2,4]triazino[5,6*-b*]indol-3-yl)thio)propanamide (44)**. The title compound was prepared from 5-methyl-5*H*-[1,2,4]triazino[5,6*-b*]indole-3-thiol (**1**) (0.050 g, 0.23 mmol) and 2-bromo*-N-*(4-chlorophenyl)acetamide (**37**) (0.064 g, 0.23 mmol) according to general procedure E. The crude product was purified by column chromatography (petroleum ether/EtOAc 1:1) to give a gray solid (59 mg, 76%). ^1^H NMR (400 MHz, DMSO-*d*_6_) δ 8.33 (d, *J* = 7.7 Hz, 1H), 7.79 (d, *J* = 3.8 Hz, 2H), 7.69–7.40 (m, 3H), 7.36 (d, *J* = 8.0 Hz, 2H), 4.80 (q, *J* = 6.9 Hz, 1H), 3.70 (s, 3H), 3.20 (s, 3H), 1.51 (d, *J* = 6.9 Hz, 3H). ^13^C NMR (101 MHz, DMSO-*d*_6_) δ 171.18, 170.94, 166.01, 146.30, 142.40, 142.15, 141.44, 132.85, 131.49, 130.07, 129.96, 123.38, 121.91, 117.69, 111.61, 55.39, 44.54, 37.69, 27.62, 24.57, 22.19, 19.31. LCMS *m/z* calc. for C_20_H_20_ClN_5_OS [M+H]^+^: 412.9, found 412.2 with *t*_R_ 2.90 min, purity > 95%.

***N***-**(4-Chlorophenyl)-2-methyl-2-((5-methyl-5*H*-[1,2,4]triazino[5,6*-b*]indol-3-yl)thio)propanamide (42)**. 5-Methyl-5*H*-[1,2,4]triazino[5,6*-b*]indole-3-thiol (**1**) (0.100 g, 0.46 mmol) was dissolved in anhydrous DMF (15 mL) under N_2_ protection in an ice bath, followed by addition of NaH 60% dispersion in mineral oil (0.020 g, 0.51 mmol) in one portion. The mixture was stirred for 45 min with ice bath cooling followed by addition of 2-bromo*-N-*(4-chlorophenyl)-2-methylpropanamide (**35**) (0.128 g, 0.46 mmol). The mixture was then slowly warmed up to room temperature and stirred overnight. Once the reaction was finished, the mixture was poured onto sat. NH_4_Cl (100 mL) and stirred vigorously for 10 min. The resulting suspension was extracted with EtOAc (30 mL × 3). The combined organic layers were washed with brine, dried over Na_2_SO_4_, and concentrated. The crude product was purified by column chromatography (petroleum ether/EtOAc 3:1) to give a yellow solid (0.140 g, 74%). ^1^H NMR (400 MHz, DMSO-*d*_6_) δ 9.98 (s, 1H), 8.29 (dt, *J* = 7.8, 1.0 Hz, 1H), 7.85–7.62 (m, 4H), 7.45 (ddd, *J* = 8.0, 6.7, 1.5 Hz, 1H), 7.38–7.17 (m, 2H), 3.73 (s, 3H), 1.81 (s, 6H). ^13^C NMR (101 MHz, DMSO-*d*_6_) δ 172.39, 166.56, 146.24, 141.98, 141.26, 138.68, 131.44, 128.76, 127.36, 123.33, 122.25, 121.90, 117.71, 111.60, 52.77, 27.78, 26.61. LCMS *m/z* calc. for C_19_H_16_ClN_5_OS [M+H]^+^: 411.9, found 411.9 with *t*_R_ 2.99 min, purity 95%.

## Results

### Virtual Screening and Hit Identification of PqsR Inhibitors

*In silico* virtual screening was performed on the PqsR ligand binding domain (LBD) crystal structure [PDB: 4JVI; (Ilangovan et al., 2013)] using the University of Nottingham Managed Chemical Compound Collection (MCCC) containing 85,061 compounds. Molecular docking was performed using both the OpenEye docking (OpenEye Scientific Software Inc. (Hawkins et al., 2007) and Schrödinger Suite for molecular modeling (Friesner et al., 2004; Halgren et al., 2004). A cut off value of the docking score function was set to -9.0 and the resulting high scoring compounds were further examined (Friesner et al., 2004; Halgren et al., 2004). This gave a library of around 500 diverse molecules that were evaluated using *in vitro* screening in a *P. aeruginosa* PAO1-L mCTX::P_*PqsA*_-*lux* reporter assay (Diggle et al., 2011).

**Figure 2:**
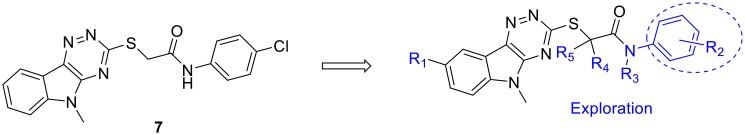
Chemical structure of hit compound **7** and plans for chemical optimisation.

Compound **7** (Figure 2) with a PAO1-L IC_50_ of 0.98 μM, was chosen for further optimisation. Disappointingly, this compound proved inactive when screened against the *P. aeruginosa* strain PA14, harboring the mCTX::P_*PqsA*_-*lux* reporter. However, the hit compound **7** provided a good chemical starting point to undertake rapid optimisation.

### Optimisation of the PqsR Inhibitor Hit

**Scheme 1:**
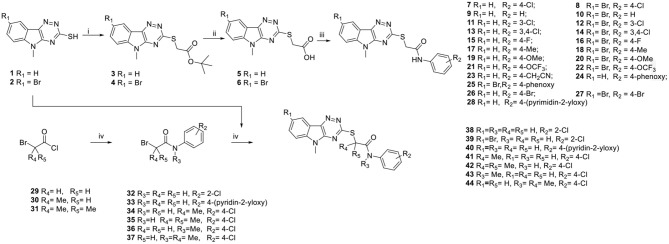
Synthesis of 2-((5-methyl-5*H*-[1,2,4]triazino[5,6-*b*]indol-3-yl)thio)acetamide derivatives. Reagents and conditions: (i) *tert-*Butyl-2-bromoacetate, NEt_3_ toluene, 0°C to rt; (ii) 4M HCl in dioxane, rt; (iii) various anilines, HATU, DMAP, NMP), rt; (iv) NEt_3_, DCM, 0°C to rt.

The chemical exploration of **7** initially focused on modifying the 4-chloroanilide, investigating the effect of substitution on the 8-position of the tricyclic ring and exploration of the effect of the core linking group substituents (groups R_3−5_). The compounds were readily synthesized according to the methods outlined below (Scheme 1).

The 1,2,4-triazino[5,6-*b*]indole-3-thiol derivatives (**1**&**2**) (Sharma et al., 2014) were functionalised in two steps through the alkylation using *tert-*butyl-2-bromoacetate and then deprotection to give the carboxylic acids (**5–6**). These key intermediates were either reacted with a wide range of anilines using HATU as the amide coupling agent to afford compounds (**7–28**) in high yield. In a complementary method, intermediates (**1**&**2**) were directly alkylated with 2-bromoacetamide intermediates (**32–37**) (Deora et al., 2019) to give compounds (**38**–**44**) in high over all yield (Scheme 1).

### Pharmacological Evaluation and Structure-Activity Relationship Studies

**Figure 3:**
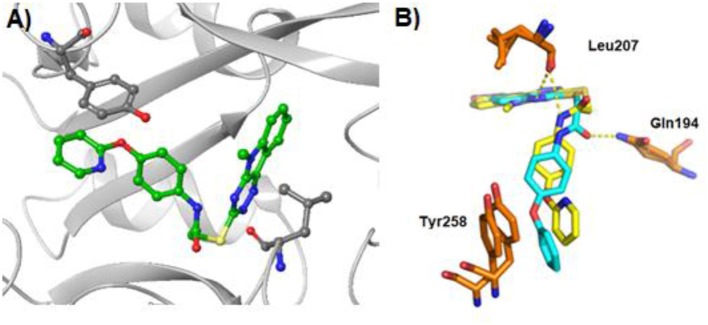
X-ray crystal structure of PqsR in complex with compound **40** and **M64** (PDB:6B8A) (Kitao et al., 2018) **(A)** X-ray co-crystal structure of **40** bound to PqsR LBD. The protein structure is presented in gray and residues Tyr^258^ and Leu^207^ are labeled in black. **(B)** Overlapping crystal structures of **40** and M64 in complex with PqsR ligand binding domain. Compound **40** is represented in yellow and M64 in light blue.

As shown in Table 1, introduction of a bromine atom to the 8-position of the 5-methyl-5*H*-[1,2,4]triazino[5,6-*b*]indole ring **8** maintained biological activity against PAO1-L and showed comparable activity against PA14. Limited SAR exploration of the core linking group showed that substitution led to a loss of biological activity (**41**–**44**). SAR exploration of the terminal aryl group proved more interesting and demonstrated differences in biological activity between the unsubstituted and 8-bromo-substituted 5-methyl-5*H*-[1,2,4]triazino[5,6-*b*]indole analogs. Removing the chlorine atom abolished biological activity (**9** and **10**), however substitution of the 4-chlorine atom with a bromine atom **26** maintained biological activity against PAO1-L and PA14. Disappointingly, removing the bromine atom (R_1_ = H) **27** led to a reduction in biological activity against PAO1-L and inactivity against PA14 (compare **26** and **27**). Moving the chlorine atom from the *para-* (**7** and **8**) to the *meta-*position (**11** and **12**) resulted in some level of biological activity against the PAO1-L strain however, **11** and **12** proved inactive against PA14. The *ortho-*chloro substituted analogs (**38** and **39**) were inactive against both strains and the 3, 4-dichloro analogs only showed activity in the 8-bromo substituted series (compare **14** vs. **13**) demonstrating divergence of SAR between the 2 series (*R*^1^ = H vs. Br). This surprising SAR was also demonstrated with the 4-fluoro analogs (**15** and **16**), where only **16** showed biological activity against the PAO1-L strain, and the 4-methyl analog **17** which was active against the PAO1-L strain (compare **17** vs. **18**). The 4-methoxy (**19** and **20**) and 4-trifluoromethoxy analogs (**21** and **22**) lost activity against both strains. Interestingly, the addition of a 4-cyanomethyl group **23** gave a compound with activity against both strains. Introduction of a phenoxy group **24** provided the activity in both strains with almost 2.5-fold higher in PAO1-L compared to **7**, although activity was again lost in the 8-bromo-5-methyl-5H-[1,2,4]triazino[5,6-b]indole series **25**. Introduction of a pyridine-2-yloxy group produced the most active compound **40** against both PAO1-L and PA14. Interestingly, increasing the number of heteroatoms on *R*_2_ **28** led to decreased PqsR inhibition. On the basis that the phenoxy group of **M64** formed a π-π stacking interaction with the side chain of Tyr^258^ (Kitao et al., 2018) and combined with docking studies, it is suggested that introducing a 6-membered heterocycle would lead to enhanced π-π stacking with Tyr^258^ in the PqsR LBD. The resulting pyridine-2-yloxy analog **40** displayed similar activity compared to **M64** at PAO1-L and an enhanced inhibition in PA14 with IC_50_ values of 0.25 ± 0.12 and 0.34 ± 0.03 μM, respectively. The proposed π-π stacking interaction between **40** and Tyr^258^ was observed by obtaining the co-crystal structure of **40** in the PqsR LBD (Figure 3).

**Table 1:** *Pseudomonas aeruginosa* P_*pqsA*_*-lux* bioreporter assays of the compounds synthesized.

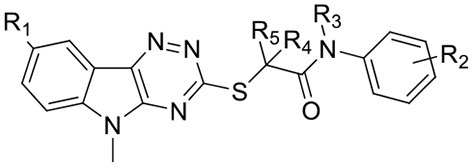							
**Example**	***R***^**1**^	***R***^**2**^	***R***^**3**^	***R***^**4**^	***R***^**5**^	**IC**_**50**_ **(μM) or Remaining Activity % (RA%) at 10 μM (bold)**	
						**PAO1-L**	**PA14**
**7**	H	4-Cl	H	H	H	0.98 ± 0.02	NA
**8**	Br	4-Cl	H	H	H	1.99 ± 0.23	1.60 ± 0.16
**9**	H	H	H	H	H	NA	NA
**10**	Br	H	H	H	H	NA	NA
**11**	H	3-Cl	H	H	H	**RA% 34 ± 7.3**	NA
**12**	Br	3-Cl	H	H	H	**RA% 43 ± 20.2**	NA
**13**	H	3,4-dichloro	H	H	H	NA	NA
**14**	Br	3,4-dichloro	H	H	H	3.1 ± 0.52	7.58 ± 0.82
**15**	H	4-F	H	H	H	NA	NA
**16**	Br	4-F	H	H	H	1.36 ± 0.21	NA
**17**	H	4-Me	H	H	H	1.86 ± 0.01	NA
**18**	Br	4-Me	H	H	H	**RA% 32 ± 6.2**	NA
**19**	H	4-OMe	H	H	H	NA	NA
**20**	Br	4-OMe	H	H	H	NA	NA
**21**	H	4-OCF_3_	H	H	H	**RA% 29 ± 6**	NA
**22**	Br	4-OCF_3_	H	H	H	**RA% 23 ± 9**	NA
**23**	H	4-(cyanomethyl)	H	H	H	0.62 ± 0.10	2 ± 0.17
**24**	H	4-phenoxy	H	H	H	0.38 ± 0.06	0.35 ± 0.06
**25**	Br	4-phenoxy	H	H	H	4.36 ± 0.42	NA
**26**	H	4-Br	H	H	H	1.71 ± 0.26	1.35 ± 0.19
**27**	Br	4-Br	H	H	H	23 ± 9	NA
**28**	H	4-(pyrimidin-2-yloxy)	H	H	H	1.04 ± 0.12	1.33 ± 0.33
**38**	H	2-Cl	H	H	H	NA	NA
**39**	Br	2-Cl	H	H	H	NA	NA
**40**	H	4-(pyridin-2-yloxy)	H	H	H	0.25 ± 0.12	0.34 ± 0.03
**41**	H	4-Cl	Me	H	H	NA	NA
**42**	H	4-Cl	H	Me	Me	NA	NA
**43**	H	4-Cl	H	Me	H	NA	NA
**44**	H	4-Cl	Me	H	Me	NA	NA
**M64**						0.32 ± 0.14	1.22 ± 0.34

*Data shown are based on n = 9. NA, not active at 10 M concentration. Bold values represent percentage of remaining activities at 10 μM concentration*.

### Crystal Structure of PqsR LBD in Complex With 40

In order to determine the mode of binding of **40** to PqsR, the co-crystal structure with the PqsR LBD was obtained at resolution of 3.2 Å (Figure 3A). The ligand-bound complex reveals that the tricyclic head group inserts deeply into the hydrophobic pocket, whilst the thioacetamide linker was positioned in the outer narrow U-shaped channel. In a similar fashion to M64, the sulfur atom on the linker allowed **40** to adopt the required conformation to fit into the binding pocket. The aromatic tail group points outside this pocket and shows a π-π stacking interaction with Tyr^258^ with a distance of 4.55 Å. Interestingly, compared to the reported crystal structure of M64, compound **40** did not establish a hydrogen bond with Gln^194^ but instead formed a hydrogen bond with Leu^207^. In addition, the pyridinyl side chain of compound **40** had an enhanced overlap with Tyr^258^ compared to the phenyl side chain of M64 (Figure 3B), which suggests that **40** could have an enhanced π-π stacking interaction with Tyr^258^ (see Supplementary Figure 1 and Supplementary Table 1 for further poses and table of crystallographic properties and refinement).

### Impact of PqsR Inhibitors on Pyocyanin Production

**Figure 4:**
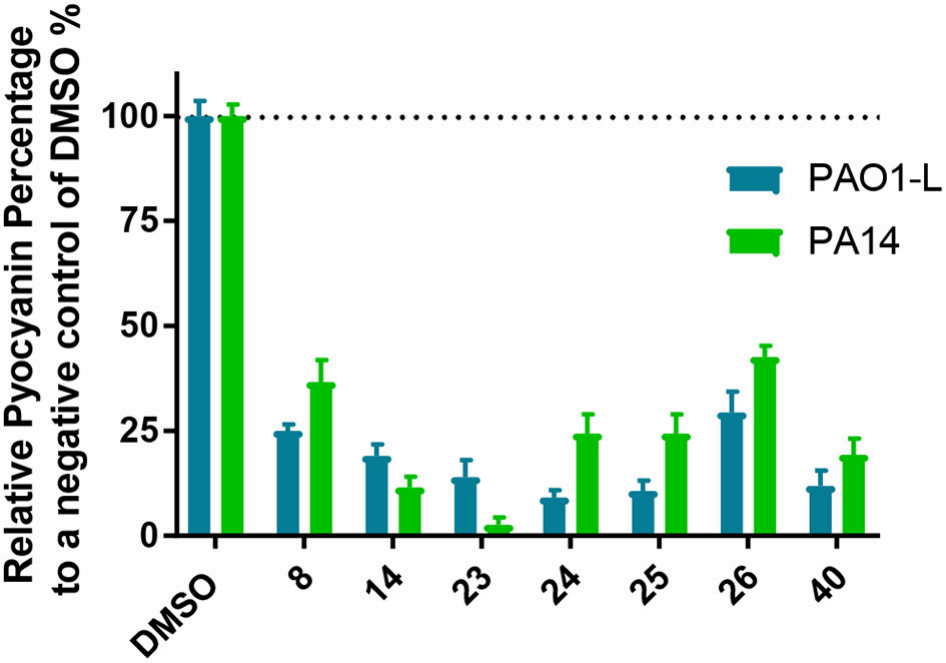
Pyocyanin production by *Pseudomonas aeruginosa* after treatment with *pqs* inhibitors **8, 14, 23, 34, 25, 26**, and **40**. Quantification of pyocyanin production after inhibitor treatment at 3 × IC_50_ for both PAO1-L and PA14. The graph represents the average of three independent experiments carried out in triplicate (*n* = *9*).

Pyocyanin is an important virulence factor for *P. aeruginosa* infections and in particular respiratory and urinary tract infections. Pyocyanin is a redox-active phenazine the production of which is regulated via PqsR and PqsE (Hall et al., 2016; Rampioni et al., 2016; Higgins et al., 2018). Therefore, pyocyanin production serves as an indirect readout of the activity of the *pqs* QS system. In this study, we measured pyocyanin expression in the presence of the most active *pqs* inhibitors in this series. The inhibitors showed a trend consistent with their corresponding IC_50_ values in the bioreporter assays. All the compounds showed inhibition of pyocyanin production of at least 50% compared with the control when used at a concentration three times higher than their IC_50_ (Figure 4). Compound **23** represented the most potent inhibitor in reducing pyocyanin production particularly in PA14. Compound **40** reduced pyocyanin production by over 80% in both strains in agreement with bioreporter assay results.

### Impact of 40 on AQ Production in Planktonic and Biofilm Cultures

**Figure 5:**
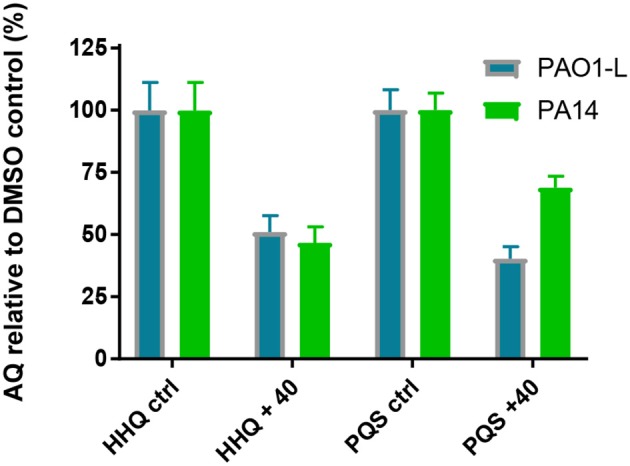
Effect of compound **40** on AQ production: Quantification of AQ concentrations after treatment with the PqsR inhibitor **40** at 3x IC_50_ value for PAO1-L and PA14 each strain. The graph represents the average of three independent experiments carried out in triplicate (*n* = *9*) for both strains.

Since PqsR controls expression of the *pqsABCDE* operon encoding the enzymes required for different stages of AQ biosynthesis, quantification of HHQ, and PQS provides a direct measurement for inhibition of the biosynthetic pathway. Compound **40** was evaluated for its effect on AQ biosynthesis at a concentration three times the corresponding IC_50_ values for PAO1-L and PA14. Inhibitor **40** was found to substantially reduce the production of HHQ and PQS in planktonic cultures of both *P. aeruginosa* strains (Figure 5).

**Figure 6:**
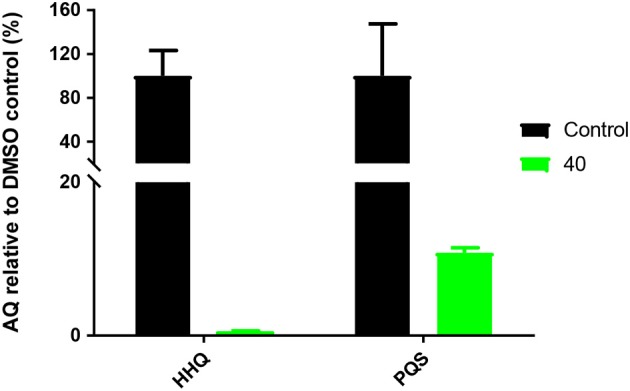
Quantification of AQ concentrations in PAO1-L biofilm cultures treated with **40** (10 μM). The y axis shows the % of AQs production in relation to a non-treated DMSO control. Biofilms were grown in M9 minimal medium for 18 h in 24-well glass bottom plates and supernatants extracted for AQ analysis.

Biofilms are a major challenge in the treatment of chronic *P. aeruginosa* infections because of their resilience and protection of bacteria from environmental stresses such as those posed by the host immune system and antimicrobial agents (Flemming et al., 2007; Rybtke et al., 2015). Many genes that are involved in biofilm formation are directly linked to *pqs* QS signaling (Rampioni et al., 2016; Lin et al., 2018). To evaluate the effect of **40** on AQ signaling in biofilm cultures, PAO1-L was grown in M9 minimal medium and challenged with either DMSO control or of **40** (10 μM). Following incubation and cell removal, supernatants were subjected to LC/MS-MS analysis. Figure 5 shows that compound **40** was able to effectively inhibit the biosynthesis of AQ signals in biofilm cultures (Figure 6).

### Effect of 40 on Biofilm Viability

**Figure 7:**
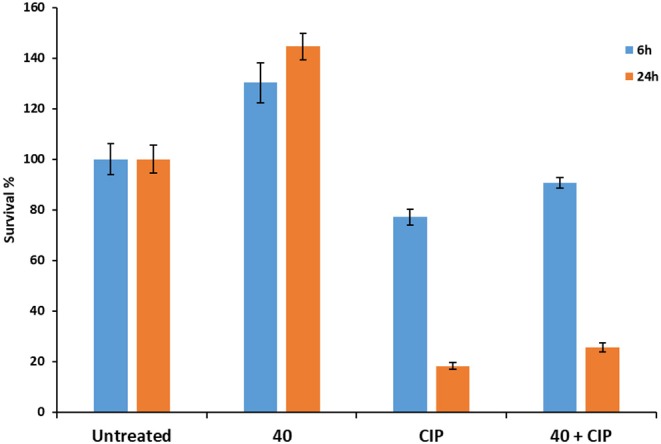
Bar chart showing PAO1-L biofilm viability quantified after treatment with different conditions for 6 or 24 h. The concentrations of the drugs used were ciprofloxacin 60 μg/mL (CIP), **40** 10 μM.

Biofilms are highly recalcitrant to the action of antimicrobials. Compound M64 has been shown to sensitize biofilms to antibiotics. To study whether **40** could also impact on biofilm formation this PqsR inhibitor was administered to pre-established PAO1-L biofilms (48 h) and their viability investigated using the LIVE/DEAD® BacLight™ bacterial viability kit and confocal laser scanning microscopy (CLSM). Moreover, to assess whether **40** can act as a non-growth inhibitory adjuvant to the broad spectrum antibiotic ciprofloxacin (CIP), combination therapy against mature PAO1-L biofilms was also tested. PAO1-L biofilms were established for 2 days before challenging with these treatments for 6 and 24 h followed by Live/Dead staining. Results showed that untreated biofilms presented only live bacteria but when the biofilms were exposed to **40** the growth of the community increased with respect to untreated controls after both 6 and 24 h (Figure 6). When the biofilms were exposed to CIP, some evidence of dead bacteria was obtained due to areas of red staining within the biofilm and, contrary to our hypothesis, treatment with the quorum sensing inhibitor **40** combined with CIP did not result in an enhanced biofilm killing (Figure 7) (see Supplementary Figures 2, 3).

## Discussion

Here, we presented the results of a hit to lead optimisation process on compound **7** (PAO1-L IC_50_ 0.98 ± 0.02 μM, PA14 inactive at 10 μM) which was discovered in an initial screening for PqsR antagonists using a bacterial cell-based reporter assay. A secondary aim of this work was to improve the potency against different *P. aeruginosa* strains. This aspect is of vital importance in antimicrobial drug discovery as the potency of inhibitors can vary widely in a strain dependent manner which has a detrimental downstream effects on their prospective success as antimicrobial agents (Jackson et al., 2018). In fact, the majority of published work in relation to PqsR inhibition has only been validated in a single strain. For this purpose, both PAO1-L and PA14 were chosen as two of the most studied laboratory strains belonging to the two major *P. aeruginosa* genomic clusters to account for any strain differences (Freschi et al., 2015).

To inform the structural variation of **7**, crystal structures with the endogenous ligand [4-hydroxy-2-heptylquinoline (NHQ)] and the inhibitor M64 were used (Ilangovan et al., 2013; Kitao et al., 2018). The PqsR LBD is dominated by lipophilic interactions, therefore the current SAR exploration mainly focused on varying the aromatic tail substituents and examining the effect of 8-position substitution. The rationale for these modifications was primarily based on molecular docking studies that provided insights into the mode of binding of **7** and approaches intended to improve potency. The substituent at the *para-* position of the aromatic ring appeared to be crucial for biological activity due to the vital π-π stacking interaction of this group with Tyr^258^. Compound **40** demonstrated almost equal sub-micromolar potency in both bioreporter strains in which a chromosomal transcriptional fusion of P_*pqsA*_-*lux* was introduced to report on PqsR activation by HHQ and PQS. These strains emit light upon activation of PqsR by the endogenous production of these signal molecules. However, upon interaction with antagonists PqsR is unable to activate the P_*pqsA*_ promoter and hence a reduction in bioluminescence is observed (Ilangovan et al., 2013; Soukarieh et al., 2018a). The results of the SAR in this assay highlight the importance of testing any prospective anti-virulence strategy on multiple genetically distinct *P. aeruginosa* strains as a means to reduce the chance of failure at both pre-clinical and clinical stages (Freschi et al., 2015).

However, compounds active in the P_*pqsA*_-*lux* reporter assay may either be inhibitors of PqsR or of the AQ biosynthetic enzymes. To confirm PqsR as the potential target for this series, the co-crystal structure of **40** with PqsR ligand binding domain was obtained. The crystal structure revealed the binding is dominated by the π-π stacking interaction with Tyr^258^ and the hydrogen bonding interaction between the amide linker of **40** and the side chain of Leu^207^, similar to that previously reported (Kitao et al., 2018).

To further confirm the inhibition of *pqs* system, the end products of this biosynthetic pathway were quantified and the inhibitory effect of **40** was clearly apparent in the phenotypic assays. The *pqs* inhibitor **40** reduced the expression of pyocyanin substantially at sub-micromolar concentrations and interfered with the biosynthesis of AQ signals leading to a significant decrease in their production in both *P. aeruginosa* genotypes corroborating the results of the bioreporter assay. Collectively, these results, along with the co-crystal structure, provide strong evidence for PqsR as the *pqs* pathway target of **40** (Zender et al., 2013; Lu et al., 2014a,b; Starkey et al., 2014). Interestingly, the inhibitory effect of **40** on AQ production extended beyond the planktonic growth of *P. aeruginosa* and was also significant in biofilm cultures where **40** drastically reduced the AQ levels. AQ signals and particularly PQS is involved in wide spectrum of functions including iron acquisition, cytotoxicity, and host immune response modulation, therefore it is of therapeutic benefit to reduce or deplete the concentration of these molecule in *P. aeruginosa* communities (Hooi et al., 2004; Diggle et al., 2007; Kim et al., 2010; Lin et al., 2018). Nevertheless, biofilm treated with **40** showed a slight increase in viability and hence growth compared to the untreated control contradicting our hypothesis. The reasons behind this observation were not investigated but may be due to multiple factors including biofilm permeability, the presence of efflux pumps or off target effects (Masi et al., 2017). Compounds with similar scaffolds were reported previously to act on PqsBC, a β-keto acyl synthase III enzyme responsible for the condensation of 2-ABA (Maura et al., 2017). Inhibition of PqsBC leads to the accumulation of 2-aminoanthranilic acid (2-AA) and 2,4-dihydroxyquinoline DHQ metabolites that increase *P. aeruginosa* persistence and promote chronic infections (Kesarwani et al., 2011; Gruber et al., 2016). Further investigation is required to ascertain the reasons behind the inability of **40** to potentiate killing by ciprofloxacin whilst being a robust inhibitor of PqsR, the production of alkylquinolones and pyocyanin.

## Data Availability Statement

The datasets generated for this study can be found in the https://www.rcsb.org/ PDB: 6TPR.

## Author Contributions

FS performed *in silico* and *in vitro* screening and directed the microbiology experiments. RL designed and performed syntheses, microbiology experiments, and contributed to writing and X-ray crystallography. FS, MS, PW, and MC designed, supervised the study and lead the writing of the manuscript. EO, MR, SR, SL, NQ, SA, SG, AM, and NH performed the experimental microbiology. WR and JE performed and designed crystallography experiments. SM, SH, TS, CB, IK-I, and RCL contributed to experimental design.

### Conflict of Interest

The authors declare that the research was conducted in the absence of any commercial or financial relationships that could be construed as a potential conflict of interest.
