# Supplementary material for: Hit Identification of New Potent PqsR Antagonists as Inhibitors of Quorum Sensing in Planktonic and Biofilm Grown Pseudomonas aeruginosa
Source: Front Chem. 2020 May 4;8:204. doi: 10.3389/fchem.2020.00204 (PMC7213079; doi:10.3389/fchem.2020.00204)
Supplement: Supplementary file 1 [file Data_Sheet_1.pdf]

Supplementary Material

(A)

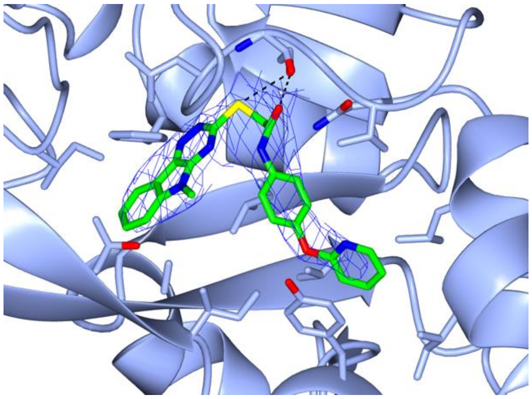

(B)

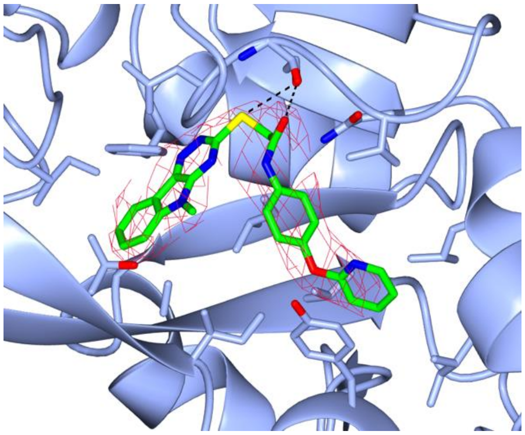

(C)

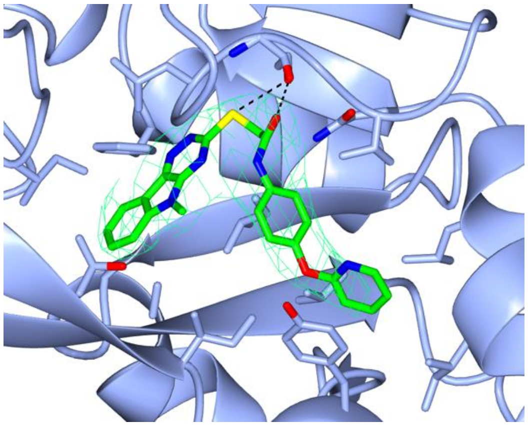

Supplementary Figure 1: Electron density map clipped to a 1.5 Å radius around compound 40. (A) 2Fo-Fc map represented as in red contoured at 1 Å (blue) (B) Standard omit map generated from by Phenix.refine contoured at 3 Å (red) (C) Polder map generated by phenix contoured at 3 Å (cyan).

Supplementary Table 1: Crystallographic table of data collection and refinement:

Data collection	
Beamline	I04-I DLS
Wavelength (Å)/beamline	0.9686
Space group	P 6_5 2 2
a, b, c (Å)	118.75, 118.75, 115.24
α, β, γ (°)	90.0, 90.0, 120.0
Resolution (Å)	76.85 – 3.2
No. of unique reflections	8390 (1466)
Rmerge (%)	23.4 (139)
Mean I/Sig(I)	6.6 (1.6)
Completeness	99.9 (99.7)

Redundnacy	13.5 (14)
CC½	0.988 (0.982)
Refinement	
Resolution range (Å)	76.85-3.20
R/Rfree (%)	0.207 / 0.242
Mean B-factor (Å)	126.16
r.m.s.d. bond lengths (Å)	0.0040
r.m.s.d. bond angles (°)	1.289
Ramachandran plot statistics (%)	
Preferred regions	96.02
Outliers	1.99
PDB ID	6TPR

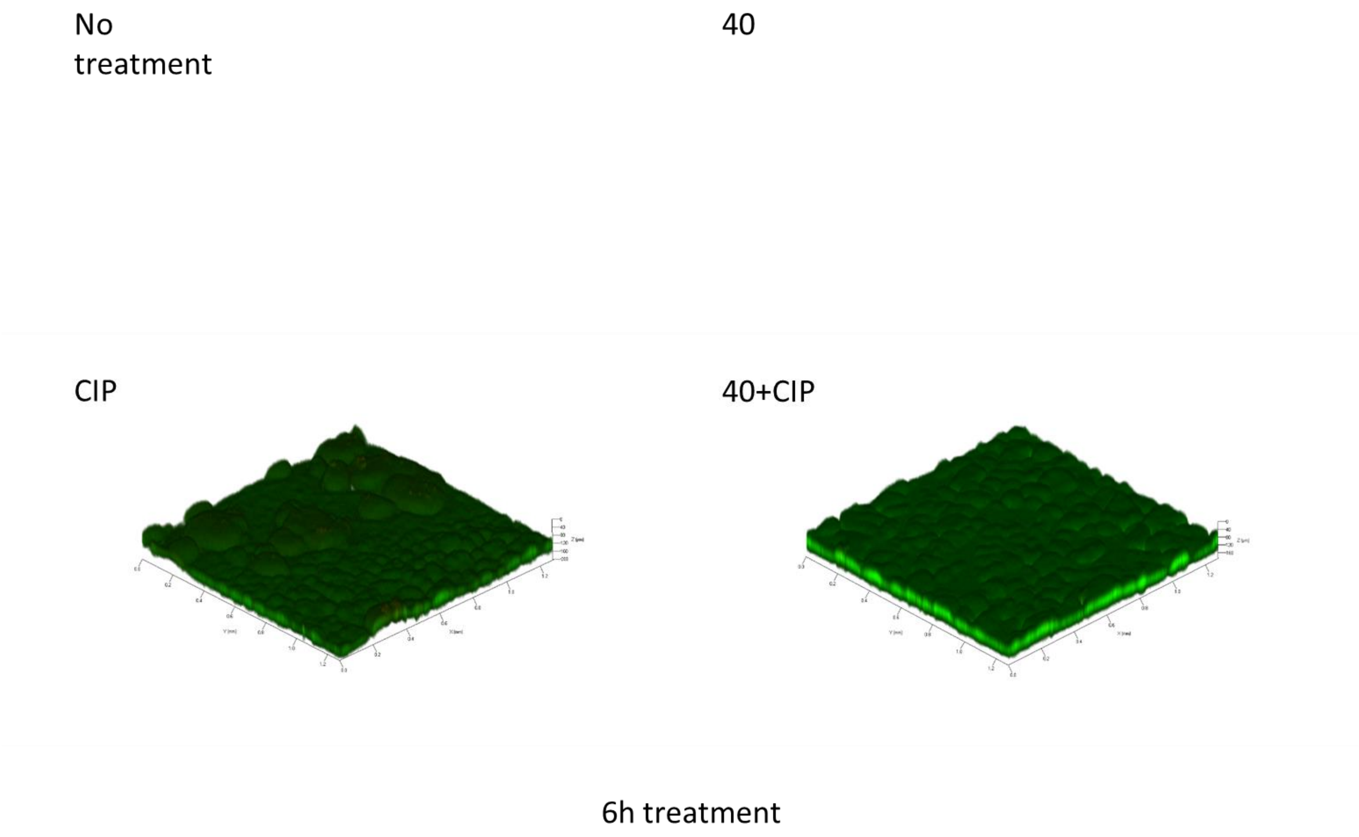

Supplementary Figure 2: Representative CLSM 3D Z-stack images PAO1-L biofilms after 2 days growth and treatment for 6 hours with: no treatment, compound 40 (10 uM), ciprofloxacin (CIP: 60µg/mL) or a combination of 40 and CIP (10 uM and 60µg/mL, respectively). The live bacteria are depicted in green (SYTO9 dye) and the dead are shown in red colour (propidium iodide stain).

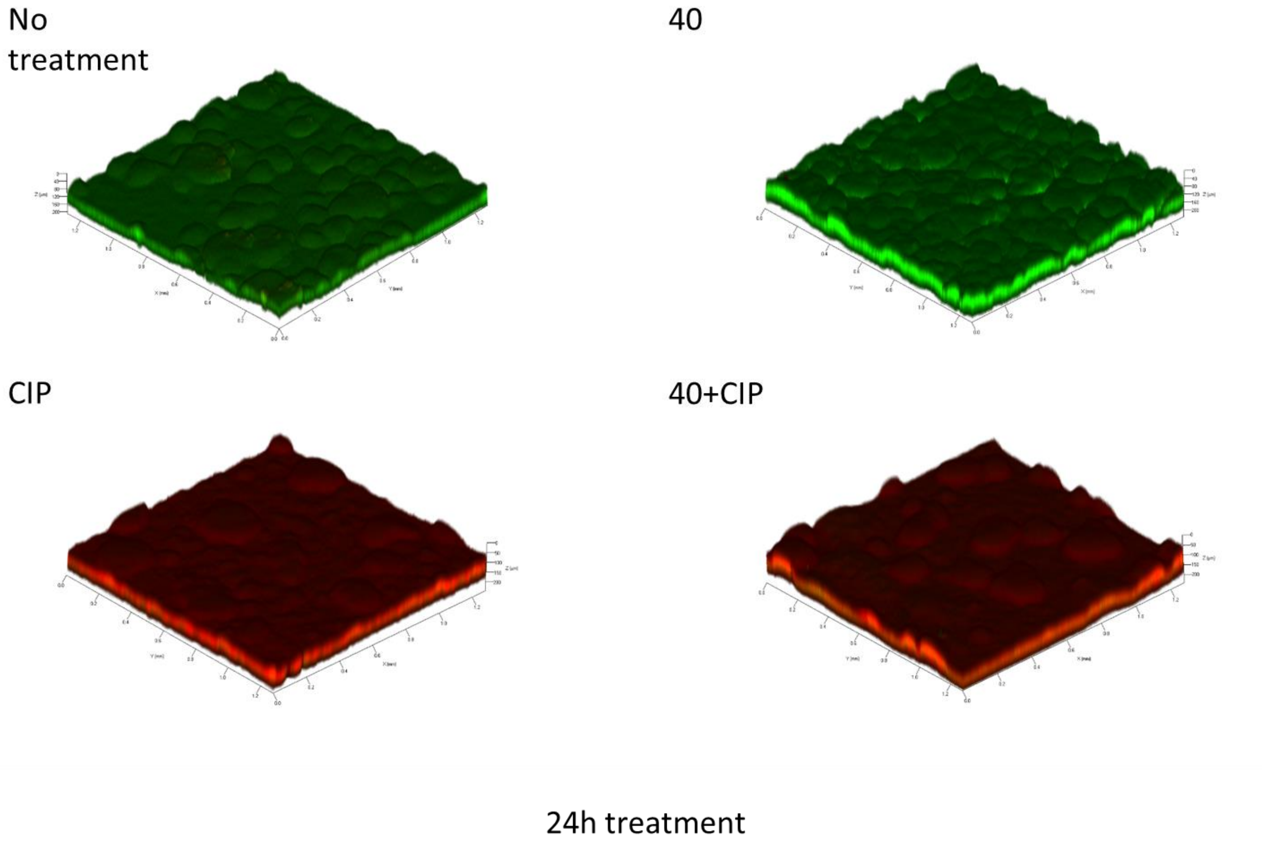

Supplementary Figure 3: Representative CLSM 3D Z-stack images PAO1-L biofilms after 2 days growth and treatment for 24 hours with: no treatment, compound 40 (10 uM), ciprofloxacin (CIP: 60µg/mL) or a combination of 40 and CIP (10 uM and 60µg/mL, respectively). The live bacteria are depicted in green (SYTO9 dye) and the dead are shown in red colour (propidium iodide stain).

Molecular docking:

Schrodinger Suite

Preparation of protein and receptor grid generation:

The X-ray crystal structure of PqsR ligand binding domain in complex with the inhibitor 3NH2-7Cl-C9QZN (PDB ID: 4JVI) was used as a protein template. The protein was prepared using the protein preparation wizard (Schrödinger Suite, version 10.7.15) where hydrogen atoms were added, water molecules were removed and the correct bond order was assigned to the amino acid residues. Afterwards, a receptor grid generation was performed based on defined residues around the ligand binding sites : (Ala102, Pro129, Ile149, Thr166, Ala 168, Val 170, Ile186, Ile189, Gln194, Ser196, Leu196, Leu197, Leu207, Leu208, Pro210, Val211, Trp234, Gly235, Pro238, Ser255). The inner grid box was set to 10 Å while the outer box was 20 Å.

Ligand preparation:

The chemical structures of the inhibitors were sketched using ChemDraw (Version 16.0.1.4) *via* an SDF file. LigPrep module was then used for final preparation of ligands into lowest energy 3D conformations. The partial atomic charges were assigned to the molecular structures using the 2005 implementation of the OPLS-AA force field. These energy minimized structures were used for Glide) docking.

Molecular Docking:

The “Extra Precision” (XP) mode of Glide docking was used to perform all docking calculations using the OPLS-AA 2005 force field. The scale factor of 1.0 for van der Waals radii was applied to atoms of protein with absolute partial charges less than or equal to 0.25. The number of position per ligand was set to five after energy minimization. The best docked structures were chosen using a XP Glide Score (XP Gscore) function as well as visual observations.

Openeye Software:

General Methods:

The three dimensional coordinates of the co-crystal structure of 3NH2-7Cl-C9QZN with $PqsR^{LBD}$ (pdb code 4JVI) was retrieved from Protein Data Bank.64 All non-protein atoms, including water were removed from the model. Using MAKE RECEPTOR 3.0.0 software (OpenEye Scientific Software) an active site model was prepared for PqsR. No constraints were used during this preparation of the receptor model. A database composed of suggested compounds for docking and the reference ligands and the newly designed compounds were processed with the Omega 2.5.1.4 software (OpenEye Scientific Software, Inc.,) to generate the lowest energy conformations for each compound. Finally, the compounds were docked into the active site models using the FRED 3.0.1 software and the Chemgauss4 scoring function (OpenEye Scientific Software, Inc., OpenEye Scientific Software: Santa Fe, NM. Http://www.eyesopen.com. April 21, 2014.). The docking was visually analyzed for the top 5 best scored poses for the conformation of the designed compounds using VIDA 4.3.0 and outputs visualized using PyMOL (The PyMOL Molecular Graphics System, Version 1.8 Schrödinger, LLC).

Microbiological Experiments:

Bacterial Strains

The *P. aeruginosa* strains and plasmids used in this study are listed in Supplementary Table 2. Bacteria were grown in (LB) at 37°C unless stated otherwise. Where required, tetracycline (Tc) was added to the media at 125 µg/ mL to select recombinants, and synthetic alkylquinolones added at the concentrations indicated.

Supplementary table 2. Bacterial strains and plasmids used in this study.

Strain or plasmid	Relevant characteristics	Reference or origin

<u>*P. aeruginosa*</u>		
PAO1-L	Wild type PAO1, Lausanne subline.	B. Holloway, via D. Haas
PAO1-L mCTX::P_{pqsA}-*lux*	PAO1-L with chromosomal mini-CTX::P_{pqsA}-*lux* insertion; Tc^R	(Soukarieh et al., 2018)
PA14	Wild type UCBPP-PA14	(Rahme et al., 1995)
PA14 mCTX::P_{pqsA}-*lux*	PA14 with chromosomal mini-CTX::P_{pqsA}-*lux* insertion; Tc^R	(Soukarieh et al., 2018)
<u>Plasmids</u>		
mini-CTX::P_{pqsA}-*lux*	R6K-based mini-CTX suicide plasmid for the chromosomal insertion of a P_{pqsA}-*lux* transcriptional reporter fusion; Tc^R	(Diggle et al., 2007)

Biosensor reporter assay:

Strains PA14 mCTX::P_{pqsA}-*lux* and PAO1-L mCTX::P_{pqsA}-*lux* were constructed using plasmid mini-CTX::P_{pqsA}-*lux* as previously described (Diggle et al., 2007) and the assay was performed according to published procedures (Fletcher et al., 2007). For screening, the compounds were tested at 10 µM concentration which was prepared from 10 mM stock in DMSO.

Pyocyanin quantification:

The experiment was performed following a published protocol with minor modification (Essar et al., 1990). *P. aeruginosa* strains were cultured into 5 mL fresh medium overnight. Compounds were assayed at 3 x IC_{50}s for 16 h at 37°C (Kuhner LT W Shaker). Cells were centrifuged at 10000 RCF for 10 min (Allegra 64R centrifuge) and the supernatant was transferred to 15 mL Falcon tubes with a HSW 10 mL Soft-Ject Syringes and a 0.22 µM Sartorius syringe-driven filters (Fisher Brand). Pyocyanin pigment was extracted into chloroform by mixing 7.5 mL of supernatant with 4.5 mL of chloroform. Pyocyanin was further extracted into 1.5 mL of 0.2 M HCL which gave a pink/red solution and the absorbance was measured at 520 nm.

LC-MS/MS

For each test sample, 100µl of sterile filtered supernatant (the same preparation as for pyocyanin) was spiked with 10µl of an internal standard solution (10µM d4-PQS in MeOH), and diluted with water to a total volume of 500µl. Samples were then extracted three times with a 0.5 mL aliquot of ethyl acetate, vortex mixing the aqueous / organic mix for 2 min, then removing the organic phase once the layers had successfully partitioned. For each sample, the combined organic extracts were dried under vacuum and re-dissolved in 100µl MeOH prior to analysis. For the LC-MS/MS analysis of supernatant extracts, the chromatography was achieved using a Shimadzu series 10AD VP LC system. The LC column, maintained at 40°C, was a Phenomenex Gemini C18 (3.0 µm, 100 x 3.0 mm) with an appropriate guard column. Mobile phase A was 0.1% (v/v) formic acid in water containing 2 mM 2-picolinic acid, and mobile phase B 0.1% (v/v) formic acid in methanol. The flow rate throughout the chromatographic separation was 450µL/min. After an injection of 2µl/sample, a binary gradient, beginning initially at 30% B, increased linearly to 99% B over 5 min. The composition remained at 99% B for 3 min, decreased to 30% B over 1 min, and stayed at this composition for 4 min to allow for column equilibration. The MS system used for analyte detection was an Applied Biosystems Qtrap 4000 hybrid triple-quadrupole linear ion trap mass spectrometer equipped with an electrospray ionisation (ESI) interface. Instrument control, data collection and analysis were conducted using Analyst software. The MS analysis was achieved with positive electrospray (+ES) multiple reaction monitoring (MRM) screening of the LC eluent for specific AQ analytes. Where chromatographic peaks for HHQ and PQS were detected, a peak area was determined and analyte peak area/internal standard peak area calculated.

AQ quantification in biofilm cultures:

Biofilms were cultivated using a 24-glass wells plate (Sensoplate, 24 well PS, F-bottom Glass Bottom). For this experiment, the *P. aeruginosa* PAO1-L strain and M9 minimal medium were used. This experiment was performed following a published protocol with several modifications (Soukarieh *et al.*, 2018). In brief, PAO1-L overnight cultures grown in LB at 37°C were diluted in fresh M9 (50 µL in 5 mL of M9) and allowed to grow until an OD_{600} of 0.5 was reached (mid-log phase). Then the cultures were normalized to OD_{600} 0.005 using M9 medium in a 24 glass-wells plate and challenged with the compounds indicated at 10 µM concentration, the final volume in each well was 1 mL and DMSO was used as solvent control. The plate was incubated for 2 h under static conditions at 37°C and then moved to a shaking incubator and grown using 60 rpm at 37°C for 18 h in order to form mature biofilm. Once the mature biofilm was obtained, all the medium in the well was removed and centrifuged at 13,000 rpm for 13 min. The supernatant was collected and the AQ extraction and quantification were performed following the LC MS/MS protocol described above.

Biofilm viability assays

Mature pre-established *P. aeruginosa* PAO1-L biofilms were grown to assess the effects of co-delivered **40** and ciprofloxacin on biofilm viability. For these assays, PAO1-L biofilms were grown on round glass coverslips (8 mm, #1.5 thickness) under flow conditions (20 rpm) in FAB containing 10

mM glucose medium inoculated with a diluted (OD600nm = 0.01) bacterial suspension from overnight cultures in LB. Biofilms were cultivated at 30°C for 2 days with medium replacement every day. Biofilms were washed in PBS to remove loosely attached cells, and incubated for a further 6 or 24 h in fresh FAB medium supplemented with **40** 10 µM or/and ciprofloxacin 60 µg/mL. Biofilms exposed to each treatment were washed in PBS and the viabilities of the attached cells were evaluated using the LIVE/DEAD® BacLight™ Bacterial Viability kit (Molecular Probes, Life Technologies) according to the manufacturer's instructions. Following staining, coverslips were rinsed with sterile water and imaged using a LSM 700 laser scanning confocal microscope (CLSM) (Carl Zeiss, Germany). Viable and non-viable biomass quantification from image stacks of biofilms was done with ImageJ software. Live/dead ratios were established for each treatment and compared with untreated controls. Five independent assays were carried out in triplicate.

Structural determination of compound 40 binding:

The recombinant PqsR$^{94\text{-}309}$ Ligand binding domain (LBD) was prepared as previously described in (Ilangovan et al., 2013). Briefly, pET28a::pqsR$^{94\text{-}309}$ was transformed into BL21(DE3) CodonPlus RIL (Agilent) with a single colony used to inoculate a 25 mL starter culture. The culture was diluted to 0.02 OD_{600} in 1 L LB and incubated at 37°C at 180 rpm. Upon reaching 0.7 OD_{600} expression was induced with 0.5 mM IPTG overnight at 18°C. Cells were harvested by centrifugation at 4000 *x g* and flash frozen. The cell pellet was thawed in 20 mM Tris-HCl (pH 7.4), 300 mM NaCl and 20 mM Imidazole (Buffer A). Lysis was achieved by sonication using an amplitude of 16 µm for 8 minutes with 15 seconds on/off cycles and lysate clarified by centrifugation at 16,000 *x g* for 1 hour. The lysate was loaded onto a HisTrap HP 5 mL column pre-charged with 0.1 M $NiSO_4$ and eluted with a 60 mL linear gradient with Buffer B containing 500 mM Imidazole. The protein was concentrated and loaded on a Superdex 75 16/60 equilibrated with 20 mM Tris-HCl (pH = 7.4), 150 mM NaCl.

A hanging drop Cryschem Plate was prepared with 100 mM Trisodium citrate, 200 mM Ammonium acetate and 2-Methyl-2,4-pentanediol (MPD). Citrate pH (5.5 – 6.5) and MPD (3%-10%) concentration was varied along the plate's X and Y axis respectively. Crystals used for soaking compound 40 were grown in conditions of 6% MPD at pH 6. For soaking the compound was dissolved in a multi-component solution consisting of MPD, Ethylene Glycol, Water and DMSO (Ciccone et al., 2015) and added directly to the drop at ten times the protein concentration. After 16-24 hours the crystal was cryocooled in a solution of 100 mM Trisodium Citrate, 200 mM Ammonium acetate, 25% MPD and 25% Glycerol. Diffraction data was collected on DLS Beamline i04 with an 0.1° oscillation at 75% transmission. Diffraction images were integrated with DIALS (Winter et al., 2018) and structure phased by Molecular Replacement with PHASER (McCoy et al., 2007) using 6Q7W (Zender et al., 2020) as a search model. Model was refined with REFAC5 (Kovalevskiy et al., 2018) with Jellybody refinement for 200 cycles prior to manual refinement in COOT (Emsley et al., 2010). Ligand restraints were generated in AceDrg (Long et al., 2017) and the ligand fitted to Fo-Fc electron density map using COOT. Structure was further refined in REMAC5 with ligand restraints and model errors resolved using geometry optimisation in Phenix.refine(Liebschner et al., 2019).

Polder and omit maps were constructed in Phenix. The final structure has been deposited in the PDB as 6TPR.

Ciccone, L., Vera, L., Tepshi, L., Rosalia, L., Rossello, A., and Stura, E.A. (2015). Multicomponent mixtures for cryoprotection and ligand solubilization. *Biotechnol Rep (Amst)* 7**,** 120-127. doi: 10.1016/j.btre.2015.05.008.

Diggle, S.P., Matthijs, S., Wright, V.J., Fletcher, M.P., Chhabra, S.R., Lamont, I.L., et al. (2007). The Pseudomonas aeruginosa 4-quinolone signal molecules HHQ and PQS play multifunctional roles in quorum sensing and iron entrapment. *Chem Biol* 14(1)**,** 87-96. doi: 10.1016/j.chembiol.2006.11.014.

Emsley, P., Lohkamp, B., Scott, W.G., and Cowtan, K. (2010). Features and development of Coot. *Acta Crystallogr D Biol Crystallogr* 66(Pt 4)**,** 486-501. doi: 10.1107/S0907444910007493.

Essar, D.W., Eberly, L., Hadero, A., and Crawford, I.P. (1990). Identification and characterization of genes for a second anthranilate synthase in Pseudomonas aeruginosa: interchangeability of the two anthranilate synthases and evolutionary implications. *J Bacteriol* 172(2)**,** 884-900.

Fletcher, M.P., Diggle, S.P., Camara, M., and Williams, P. (2007). Biosensor-based assays for PQS, HHQ and related 2-alkyl-4-quinolone quorum sensing signal molecules. *Nat Protoc* 2(5)**,** 1254-1262. doi: 10.1038/nprot.2007.158.

Ilangovan, A., Fletcher, M., Rampioni, G., Pustelny, C., Rumbaugh, K., Heeb, S., et al. (2013). Structural basis for native agonist and synthetic inhibitor recognition by the Pseudomonas aeruginosa quorum sensing regulator PqsR (MvfR). *PLoS Pathog* 9(7)**,** e1003508. doi: 10.1371/journal.ppat.1003508.

Kovalevskiy, O., Nicholls, R.A., Long, F., Carlon, A., and Murshudov, G.N. (2018). Overview of refinement procedures within REFMAC5: utilizing data from different sources. *Acta Crystallogr D Struct Biol* 74(Pt 3)**,** 215-227. doi: 10.1107/S2059798318000979.

Liebschner, D., Afonine, P.V., Baker, M.L., Bunkoczi, G., Chen, V.B., Croll, T.I., et al. (2019). Macromolecular structure determination using X-rays, neutrons and electrons: recent developments in Phenix. *Acta Crystallogr D Struct Biol* 75(Pt 10)**,** 861-877. doi: 10.1107/S2059798319011471.

Long, F., Nicholls, R.A., Emsley, P., Graaeulis, S., Merkys, A., Vaitkus, A., et al. (2017). AceDRG: a stereochemical description generator for ligands. *Acta Crystallogr D Struct Biol* 73(Pt 2)**,** 112-122. doi: 10.1107/S2059798317000067.

McCoy, A.J., Grosse-Kunstleve, R.W., Adams, P.D., Winn, M.D., Storoni, L.C., and Read, R.J. (2007). Phaser crystallographic software. *J Appl Crystallogr* 40(Pt 4)**,** 658-674. doi: 10.1107/S0021889807021206.

Rahme, L.G., Stevens, E.J., Wolfort, S.F., Shao, J., Tompkins, R.G., and Ausubel, F.M. (1995). Common virulence factors for bacterial pathogenicity in plants and animals. *Science* 268(5219)**,** 1899-1902.

Soukarieh, F., Vico Oton, E., Dubern, J.F., Gomes, J., Halliday, N., de Pilar Crespo, M., et al. (2018). In Silico and in Vitro-Guided Identification of Inhibitors of Alkylquinolone-Dependent Quorum Sensing in Pseudomonas aeruginosa. *Molecules* 23(2). doi: 10.3390/molecules23020257.

Winter, G., Waterman, D.G., Parkhurst, J.M., Brewster, A.S., Gildea, R.J., Gerstel, M., et al. (2018). DIALS: implementation and evaluation of a new integration package. *Acta Crystallogr D Struct Biol* 74(Pt 2)**,** 85-97. doi: 10.1107/S2059798317017235.

Zender, M., Witzgall, F., Kiefer, A., Kirsch, B., Maurer, C.K., Kany, A.M., et al. (2020). Flexible Fragment Growing Boosts Potency of Quorum-Sensing Inhibitors against Pseudomonas aeruginosa Virulence. *ChemMedChem* 15(2)**,** 188-194. doi: 10.1002/cmdc.201900621.
